# Activating cGAS–STING axis contributes to neuroinflammation in CVST mouse model and induces inflammasome activation and microglia pyroptosis

**DOI:** 10.1186/s12974-022-02511-0

**Published:** 2022-06-10

**Authors:** Rui Ding, Haiyan Li, Yaqi Liu, Weiyang Ou, Xifang Zhang, Huihui Chai, Xiaofei Huang, Weijie Yang, Qiujing Wang

**Affiliations:** 1 Department of Cerebrovascular Surgery, The Third Affiliated Hospital of Sun Yat-Sen University, No 600 Tianhe Road, Guangzhou, 510630 Guangdong China; 2 Department of Neurology, The Third Affiliated Hospital of Sun Yat-Sen University, No 600 Tianhe Road, Guangzhou, Guangdong China; 3 The National Key Clinical Specialty, The Engineering Technology Research Center of Education Ministry of China, Guangdong Provincial Key Laboratory On Brain Function Repair and Regeneration, Department of Neurosurgery, Zhujiang Hospital, Southern Medical University, Guangzhou, 510282 Guangdong China; 4 Dongguan Kanghua Hospital, 1000# Dongguan Avenue, Dongguan, 523000 Guangdong Province China

**Keywords:** cGAS, STING, Neuroinflammation, NLRP3 inflammasome, Microglia pyroptosis

## Abstract

**Background:**

Neuroinflammation-induced injury is intimately associated with poor prognosis in patients with cerebral venous sinus thrombosis (CVST). The cyclic GMP-AMP synthase–stimulator of interferon gene (cGAS–STING) axis is a cytoplasmic double-stranded DNA (dsDNA) sensing pathway has recently emerged as a crucial mediator of neuroinflammation in ischemic stroke. However, the role of the cGAS–STING pathway in modulating post-CVST inflammation and the underlying mechanisms involved remain unclear.

**Methods:**

A CVST model was induced by ferric chloride in male C57BL/6J mice. The selective cGAS inhibitor RU.521, STING agonist 2′3′-cGAMP, and STING siRNA were delivered by intranasal administration or intraventricular injection. Post-CVST assessments included rotarod test, TUNEL staining, Fluoro-Jade C staining, dihydroethidium staining, western blotting, qPCR, immunofluorescence, immunohistochemistry, ELISA and flow cytometry.

**Results:**

cGAS, STING, NLRP3 and GSDMD were significantly upregulated after CVST and mostly in the microglia of the mouse brain. CVST triggered the release of dsDNA into the cytoplasm and elicited an inflammatory response via activating the cGAS–STING axis. RU.521 decreased the levels of 2′3′-cGAMP, STING and downstream inflammatory cytokines, and suppressed the expressions of NLRP3 inflammasome and pyroptosis-pertinent components containing cleaved caspase-1, GSDMD, GSDMD-C, pro- and cleaved IL-1β, and cleaved IL-1β/pro-IL-1β. Besides, RU.521 treatment also reduced oxidative stress, lessened the numbers of microglia and neutrophils, and ameliorated neuronal apoptosis, degeneration along with neurological deficits post-CVST. 2′3'-cGAMP delivery enhanced the expressions of STING and related inflammatory mediators, NLRP3 inflammasome and pyroptosis-relevant proteins, whereas these alterations were significantly abrogated by the silencing of STING by siRNA.

**Conclusions:**

Our data demonstrate that repression of the cGAS–STING pathway diminishes the neuroinflammatory burden of CVST and highlight this approach as a potential therapeutic tactic in CVST-mediated pathologies.

**Supplementary Information:**

The online version contains supplementary material available at 10.1186/s12974-022-02511-0.

## Introduction

Cerebral venous sinus thrombosis (CVST) is an underrated and potentially fatal cause of stroke with a reported mortality of 5–10% worldwide. The impaired venous drainage attributed to thrombus obstruction largely leads to ischemia, cerebral edema, and parenchymal lesions that can be complicated by infarction or hemorrhage reported in approximately 60% of cases [1–3]. Unfortunately, in addition to anticoagulant and thrombolytic therapy, effective treatments targeting the damaged brain parenchyma after CVST remain limited to date [2]. Previously, reliable evidences have shown that inflammation and the immune response occur minutes to hours after CVST. These events can last for at least 14 days post-CVST and act to enhance secondary neuro-immunologic deterioration and aggravate parenchymal lesions and so may be potential strategies for therapeutic intervention [4–6].

Cyclic GMP-AMP synthase (cGAS, also known as MB21D1) is a crucial cytoplasmic DNA receptor that recognizes endogenous and exogenous double-stranded DNA (dsDNA, a key damage-related molecular pattern (DAMP)) [7, 8]. cGAS then catalyzes a reaction between guanosine triphosphate (GTP) and adenosine triphosphate (ATP) to produce a secondary messenger called cyclic GMP-AMP (2′3′-cGAMP). 2′3′-cGAMP is an endogenous ligand that binds and activates the key adaptor protein stimulator of interferon gene (STING) which mediates the production of inflammatory factors (e.g., TNF-α, IL-6, MCP-1) by activating transcription factor NF-κB to exacerbate the inflammatory response and immune imbalance [8–10].

Noticeably, activation of the cGAS–STING pathway triggered by the abnormal accumulation of dsDNA in the cytoplasm has been demonstrated in multiple diseases including amyotrophic lateral sclerosis [11], neonatal hypoxic-ischemic encephalopathy [12], and cerebral ischemia/reperfusion injury [9]. The pharmacologic inhibition or genetic deletion of cGAS and its partner STING could efficiently alleviate inflammation-relevant damage and ameliorate neuropathology. Likewise, after the ictus of CVST, abundant dsDNA may be released by necrotic neuronal cells. The potential mechanisms of cerebral inflammation on aberrant dsDNA recognition via the cGAS–STING axis following CVST are yet to be investigated.

Inflammasomes are intracellular multiprotein scaffolds that assemble in response to the pathogen- or damage-associated stimuli during the inflammatory and immune responses [13, 14]. The Nod-like receptor family pyrin domain-containing 3 (NLRP3) inflammasome is the most thoroughly investigated inflammasome. It comprises a sensor (NLRP3), an adaptor (ASC), and an effector (pro-inflammatory precursor pro-caspase-1), which exerts pivotal roles in various types of stroke including CVST. Activation of the NLRP3 inflammasome hinges on two functionally disparate priming and activation steps [13, 15, 16]. The priming step involves the activation of nuclear factor NF-κB, which contributes to the synthesis of pro-IL-1β and NLRP3, whereas the activation step was tightly related to the production of reactive oxygen species (ROS) [17] and relevant molecules (e.g., thioredoxin-interacting protein (TXNIP) [18]), leading to NLRP3 inflammasome assembly followed by caspase-1 cleavage. Intriguingly, it has been shown that STING also engages in the priming or activation of the NLRP3 inflammasome via NF-κb [19] and other mechanisms (e.g., potassium efflux [20] and deubiquitination [21]).

Pyroptosis is a pro-inflammatory type of programmed cell death that relies on the activity of cytosolic gasdermin-D (GSDMD) driven by the NLRP3 inflammasome and the like [14, 22, 23]. Specifically, active caspase-1 cleaves GSDMD into the N-terminal (GSDMD-N) and the autoinhibitory C-terminal (GSDMD-C) [24]. Also, GSDMD-N binds to the plasmalemma and oligomerizes to form membrane pores, sequentially leading to the release of abundant pro-inflammatory cytokines (IL-1β and IL-18) which further boost the recruitment of immune cells and exacerbate the inflammatory cascade [25].

Microglia are crucial mediators of neuroinflammation and the immune response following central nervous system (CNS) injury [26, 27], and are also regarded as the main cell type in which pyroptosis occurs in the CNS [9, 23, 28]. Momentously, NLRP3 inflammasome-mediated GSDMD-triggered pyroptosis in microglia has been implicated in the pathogenesis of subarachnoid hemorrhage [29], cerebral ischemia/reperfusion injury [30, 31] and spinal cord injury [23]. Although microglia pyroptosis is repeatedly delineated in distinct neuroinflammatory-related diseases, the underlying mechanism of this process remains to be fully elucidated in CVST. Recent evidence has demonstrated that activation of the cGAS–STING axis in response to cytosolic DNA stimulation engaged in NLRP3 inflammasome activation [20, 21] and GSDMD-triggered microglia pyroptosis [9]. Nonetheless, the interplay among the cGAS–STING axis, the NLRP3 inflammasome, and microglia pyroptosis post-CVST remains to be determined.

In the present study, we demonstrated that the cGAS–STING axis was upregulated in a mouse model of CVST. Blocking cGAS with RU.521 (a potent and selective inhibitor of cGAS [12, 32]) suppressed the inflammatory cascade and NLRP3 inflammasome-mediated microglia pyroptosis. Also, the CVST-mediated inflammatory pathologies were aggravated by delivery of the STING agonist 2′3'-cGAMP and that these alterations were remarkably abolished by STING silencing using siRNA.

## Materials and methods

### Animals

A total of 201 male C57BL/6 J mice (8–10 weeks old, 22–25 g) were obtained from the Animal Experiment Center of Sun Yat-Sen University. Animals were housed in a specific pathogen-free laboratory with fixed 12-h light/dark cycles under controlled temperature and humidity conditions (22 ± 1 °C; 45–55%). Standard rodent food and water were available ad libitum throughout the whole experiment. All animal protocols were approved by the Sun Yat-sen University Ethics Committee in conformity to the guidelines for the Care and Use of Laboratory Animals by the National Institutes of Health (Approval No. L102012018040Z).

### Induction of superior sagittal sinus (SSS) thrombosis

Modeling of SSS thrombosis was performed as previously described with some modifications [4, 33]. Briefly, each mouse was anesthetized by intraperitoneal injection of sodium pentobarbital (40 mg/kg) and fixed in a stereotactic frame. The skull was exposed at the dorsal aspect of the head by a median skin incision of 1.5 cm. After the bregma and lambda were identified, a high-speed drill was employed to create a longitudinal cranial window between them under a surgical microscope (Carl Zeiss, Inc., Wetzlar, Germany). The drill tip was cooled frequently with physiological saline to alleviate thermal damage. The SSS was carefully exposed to ensure that the sinus and dura remained intact. Thrombosis of the sinus was induced by topical placement of a 5.5 mm long 5–0 silk thread (orientated from the site of the lambda to the site of bregma) soaked with 20% ferric chloride (FeCl_3_, 157740, Sigma-Aldrich) solution on the surface of SSS for 6 or 12 min. If a sufficient thrombus to cause complete blockage of SSS was not produced within 6 min, the FeCl_3_ attachment was repeated for a further 6 min. After the attachment of the ferric chloride, the field was flushed several times with warm saline. Adjacently, the incision was sutured and the mouse was subcutaneously injected with 0.2 mL of saline to prevent dehydration. In sham treated animals, a saline-soaked silk thread was applied to the SSS. Throughout the surgical procedure, the body temperature was sustained at 37 °C using a thermostatic heating pad.

### Experimental group design (Additional file 1: Fig. S1)

#### Experiment 1

36 mice were randomized to six groups consisting of a sham group and five CVST subgroups (at 6 h, 1 d, 3 d, 7 d, and 14 d post-CVST, n = 6). Samples of mouse brain tissues were collected for the Western blot and qPCR detection of the dynamic patterns of diverse proteins. Besides, 9 mice were assigned to the sham group, 1d, and 3d after CVST (n = 3 per group) for immunofluorescence staining.

#### Experiment 2

122 mice were randomized into three groups including a sham + vehicle (1% DMSO + corn oil) group, a CVST 3d + vehicle group and a CVST 3d + RU.521 group. Motor function (n = 6, 8, 9 in the three groups, respectively) was assessed by the rotarod test. The samples were collected and analyzed by DHE staining (n = 4), terminal deoxynucleotidyl transferase dUTP nick-end labeling (TUNEL), Fluoro-Jade C (FJC), immunofluorescence (IF) and immunohistochemistry (IHC) staining (n = 6), Western blot (WB, n = 6), enzyme-linked immunosorbent assay (ELISA, n = 6), qPCR (n = 6) and flow cytometry (n = 5).

#### Experiment 3

34 mice were randomized into the four groups containing a CVST 3d + vehicle (phosphate-buffered saline, PBS), a CVST 3d + 2′3′-cGAMP group, a CVST 3d + Scramble siRNA group and a CVST 3d + STING siRNA group. Samples were collected and analyzed by TUNEL and FJC staining (n = 5), and WB (n = 6).

### Drug administration

RU.521 (450 ug/kg, RU5-42-01, InvivoGen, USA) was dissolved in 1% DMSO + corn oil (DMSO, Sigma-Aldrich; corn oil, Cat.No.: HY-Y1888. MedchemExpress) and intranasally administrated at 2 h, 12 h, 24 h post-CVST, and then once a day until the seventh day. 2′3′-cGAMP (500 μg/kg, GA3-42-03, InvivoGen, USA) or vehicle (phosphate-buffered saline, PBS) was intranasally administrated at 2 h, 12 h, 24 h, and 48 h after CVST. STING siRNA (320 pmol, E-055528-00-0005, Dharmacon, USA) and scrambled siRNA (320 pmol, D-001910-01-05, Dharmacon, USA), dissolved in 2 ul of sterile DNase/RNase-free water, were slowly injected into the right lateral ventricle with the aid of a stereotaxic frame (the coordinates relative to bregma: 0.5 mm posterior, 1.0 mm lateral, 2.25 mm deep from the skull surface) at 48 h before CVST induction. The drug-delivery way and dosage mentioned were selected as previously described [12].

### Preparation of paraffin and frozen sections

**Fig. 1:**
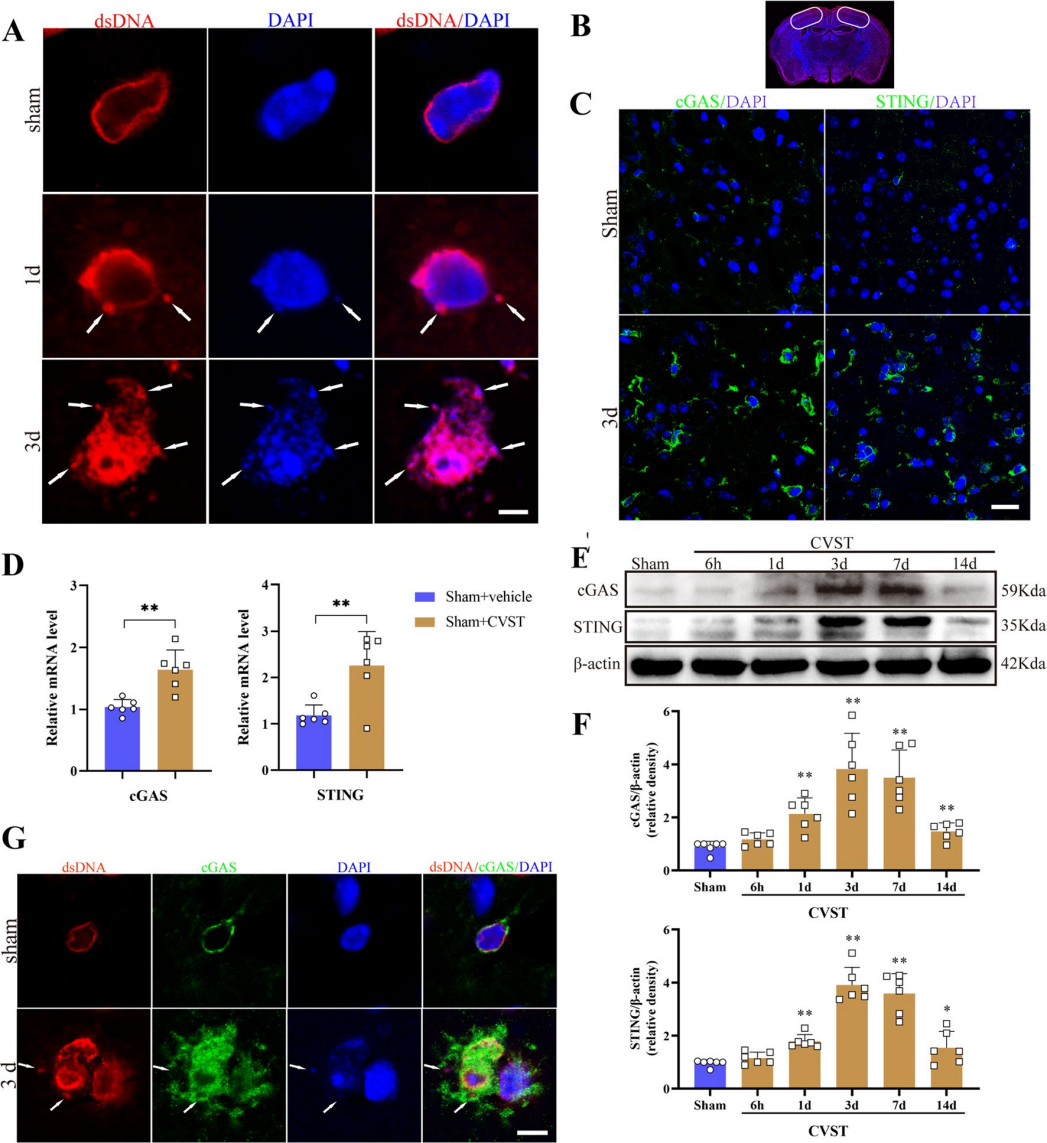
Accumulation of double-strand DNA (dsDNA) and dynamic changes of cGAS and STING after CVST. **A** Typical immunofluorescent staining for dsDNA at the sham group, and 1 d and 3 d post-CVST, white arrowheads hint disintegration of the nucleus. Scale bar = 5 um. **B** A schematic indicating the selected brain cortex region for the whole experiment (white frame). **C** Representative immunofluorescent staining of cGAS and STING in the sham group and 3 d after CVST. Scale bar = 25 um. **D** The mRNA expression levels of the cGAS and STING post-CVST were measured by qPCR. n = 6 mice per group. **P < 0.01. **E** Western blot assay for the temporal profile of cGAS and STING levels from the injured cortex at sham, 6 h, 1 d, 3 d, 7 d and 14 d post-CVST. **F** Quantitative analysis for Western blot. n = 6 mice per group. **P < 0.01, *P < 0.05. **G** Representative microphotographs of immunofluorescence double staining showing the relationship between dsDNA (red) with cGAS (green) in the sham group and CVST 3 d group. Scale bar = 10 μm

After deep anesthesia, mice were transcardially perfused with saline followed by 4% paraformaldehyde at specific time points after CVST. For paraffin slices, after fixation in 4% paraformaldehyde, dehydration, and paraffin-embedding, 4 μm-thickness coronary sections of the mouse brain (as shown in Fig. 1B) samples were prepared. For frozen sections, perfused brain tissues were immersed in 4% paraformaldehyde at 4 ℃ overnight and then incubated in 15 and 30% (wt/vol) sucrose in PBS for 24 h at 4 °C. Mouse brains were then embedded in the optimal cutting temperature (O.C.T.) compound and stored at − 80 °C. Consecutive 10-um-thick coronal sections were prepared using a freezing microtome (CM1950, Leica, Germany) at temperatures below − 20 °C.

### TUNEL and FJC staining

Apoptosis was detected with a terminal deoxynucleotidyl transferase dUTP nick-end labeling (TUNEL) kit (C1090, Beyotime, China) according to the manufacturers instructions. Briefly, paraffin-embedded brain sections were dewaxed and incubated with Proteinase K (20 μg/mL) for 20 min at room temperature. After being washed with PBS, sections were incubated with the TUNEL reaction mixture for 60 min at 37 °C in the dark. Nuclei were counterstained with DAPI (C1005, Beyotime) and the sections were visualized and captured under a Leica fluorescent microscope (DM2500, Germany) and a Nikon microscope (TI2-E). TUNEL‐positive cells were expressed as a percentage of the total cell count.

Fluoro-Jade C (FJC) staining was performed using a commercial detection kit (Millipore, Darmstadt, Germany) to evaluate degenerating neurons as previously depicted [34]. Data are presented showing the average number of FJC-positive neurons in the fields as cells/mm^2^. The number of stained cells was analyzed using Image J software.

### Dihydroethidium (DHE) staining

DHE staining was employed to detect the ROS levels in mouse brains as previously reported [34]. Fresh frozen brain Sects. (10 um) were incubated with 2 μmol/L fluorescent dye dihydroethidium (D7008, Sigma-Aldrich, Germany) at 37 °C for 30 min in a dark humidified chamber. After counterstaining with DAPI for 10 min, images of the ROS staining were captured via a fluorescence microscope (Leica, DMI8, Germany). For the quantitative analysis, the average of the DHE-positive cells was calculated from two randomly selected areas with the use of ImageJ software (ImageJ, USA) and expressed as a percentage of the total cell count.

### Rotarod test

The post-CVST motor functions were investigated using the rotarod test as formerly described [35]. Briefly, mice were placed on a rotating drum with a speed accelerating from 4 to 40 rpm within 5 min. The times at which the mice fell off the drum (latency to fall) were recorded. Before surgery, 5 trials were carried out on each mouse and the mean time of trial numbers 3, 4, and 5 was used as the pre-surgery baseline. After the operation, mice were tested for 5 trials on the 1 d, 3 d, 5 d, 7 d, 10 d, 14 d post-CVST with intervals of 5 min at least. The data for trial numbers 3–5 were utilized to calculate the mean time of latency to fall.

### IF staining

Paraffin sections were prepared as aforementioned and antigen retrieval was performed with Citrate–EDTA Antigen Retrieval Solution (P0086, Beyotime, China) in a microwave for 25 min. The slides were then blocked with 5% goat serum (SL038, Solarbio life sciences, china) at room temperature for 30 min and then incubated overnight at 4 °C with primary antibodies as follows: rabbit anti-STING antibody (1:100, NBP2-24683SS, Novus), rabbit anti-GSDMD antibody (1:500, ab219800, Abcam), mouse anti-IBA-1 antibody (1:200, GB12105, Servicebio), mouse anti-NeuN antibody (1:500, ab104224, Abcam), mouse anti-GFAP antibody(1:200, GB12105, Servicebio), rabbit anti-TNF-α antibody (1:200, GB11188, Servicebio). Additionally, the IF staining of the frozen slices was conducted as previously described [9]. Briefly, after fixation, permeabilization, and blocking, the sections were incubated with the following primary antibodies at 4 °C overnight: mouse anti-dsDNA (sc-58749, 1:100, Santa Cruz Biotechnology), rabbit anti-cGAS antibody (GTX02874, 1:100, Gentex), mouse anti-8-OhdG antibody (1:300, ab62623, Abcam).

For immunofluorescent double-staining, the primary antibodies were mixed and incubated under the same conditions. The sections were washed with PBS and then incubated with the corresponding secondary antibodies as follows: goat anti-mouse IgG H&L (Alexa Fluor® 594), 1:400, Abcam, ab150116; goat anti-rabbit IgG H&L(Alexa Fluor® 488), 1:400, Abcam, ab150077; Goat Anti-Mouse IgG H&L (Alexa Fluor® 488), 1:400, Abcam, ab150113; Goat Anti-Rabbit IgG H&L (Alexa Fluor® 594),1:400, Abcam, ab150080) for 1 h at 37 ℃. After washing with PBS, the nuclei were counterstained with DAPI (C0065, Solarbio, China) for 10 min. Images were captured by fluorescence (Leica, DMI8, Germany) and confocal microscopy (Leica, TSCSP8, Germany).

### IHC staining

IHC staining was performed according to the commercial kits (PV-6001 and PV-6002, ZSGB-Bio, Beijing, China). Antigen retrieval of the paraffin sections was performed using the same protocol described for IF staining. Subsequently, sections were incubated with 0.3% H_2_O_2_ for 10 min to inactivate endogenous peroxidase activity and then washed with PBS. After being blocked with 5% goat serum for 15 min, slices were incubated overnight at 4 °C with the primary antibodies as follows: rabbit anti-NLRP3 antibody (ab214185, 1:200, Abcam), rabbit anti-cleaved-caspase 1 p20 (1:100, AF4005, Affinity), IL-1β (1:100, GTX74034, Gentex), rabbit anti-GSDMD (1:800, ab219800, Abcam), mouse anti-IBA-1 (1:300, GB12105, Servicebio), rabbit anti-Ly6G (1:500, GB11229, Servicebio), rabbit anti-CD68 (1:100, DF7518, Affinity). The sections were incubated with enzyme-conjugated goat anti-mouse IgG or goat anti-rabbit IgG polymer for 30 min. Finally, immunoreactivity was visualized using 3,3-diaminobenzidine (DAB, ZLI-9017, ZSGB-Bio) followed by restaining with hematoxylin. Images were captured by a light microscope (Leica, DM2500, Germany).

### Western blot analysis (WB)

After transcardial perfusion with pre-cooling saline, brain tissue samples from the injured cortex were immediately harvested and homogenized with a freezing grinding mill (70 Hz, 60 s, LUKA, Guangzhou). Total protein was isolated and extracted according to the protocol of a protein extraction kit (BC3711, Solarbio, China). Protein concentrations were measured using a BCA Protein Assay Kit (PC0020, Solarbio). Subsequently, 30 ug of protein sample was separated by 6–15% SDS-PAGE and then transferred to PVDF membranes. The PVDF membranes were blocked in 5% skimmed milk or BSA for 2 h at room temperature and incubated at 4 ℃ overnight with primary antibodies as follows: rabbit anti-cGAS (#31659, 1:1000, CST), rabbit anti-STING (1:1000, CST, #13647), rabbit anti-NLRP3 (1:1000, #15101, CST), rabbit anti-GSDMD (1:1000, #93709, CST), rabbit-anti-cleaved C-terminal GSDMD (ab255603,1:1000, Abcam), rabbit anti-TXNIP (1:1000, ab188865, Abcam), rabbit anti-p-NF-kb p65(Ser536) (1:1000, AF2006, Affinity), rabbit anti-NF-kb p65 (1:1000, ab16502, Abcam), mouse anti-MCP-1 (1:1500, 66272-1-Ig, Proteintech), caspase-1 p20 (1:500, sc-398715, Santa Cruz), IL-1β (1:100, GTX74034, Gentex) and HRP-conjugated β-actin mouse monoclonal antibody (1:5000, HRP-66009, Proteintech).

After being rinsed with PBS, the PVDF membranes were incubated with secondary antibodies (Goat Anti-Rabbit IgG(H + L), 1:10,000, CW0103, CWBIO, China, or Goat Anti-Mouse IgG(H + L), 1:10,000, CW0102, CWBIO) for 1 h at room temperature. Finally, the blots were visualized employing a Millipore ECL kit and a gel imaging system (Tanon, 5200SF, Shanghai, China). Protein expression was quantified using ImageJ 1.5 software (National Institutes of Health, USA).

### ELISA

The cerebral cortex tissues of mice were harvested and homogenized, consistent with the method of western blot. The protein concentrations were detected using a BCA Protein Assay Kit (MM-9227B). The levels of cerebral oxidative damage biomarkers including 8-hydroxy-2-deoxyguanosine (8-OHdG), 3-nitrotyrosine (3-NT) and malondialdehyde (MDA) were determined utilizing assay kits for 8-OHdG (MM-0221M2), 3-NT (MM-45185M2) and MDA (MM-0897M2) according to the manufacturer’s instructions. The levels of 2′3′-cGAMP were detected by a 2′3′-cGAMP assay Kit (MM-44862M2) in conformity to the manufacturer’s instructions. All the kits are purchased from Jiangsu Meimian Industrial Co., Ltd. China.

### Quantitative real-time polymerase chain reaction (qPCR) analysis

Total RNA was isolated from homogenized cerebral cortex tissues using Trizol reagent (R1100, Solarbio) and quantified using a NanoDrop 2000 spectrophotometer (Thermo Scientific, Bremen, Germany). Reverse transcription was performed with a primescript RT reagent kit (AG11728, Accurate Biology) according to the manufacturer’s instructions. Real-time RT-PCR was performed in a total volume of 10 μL containing 1 μL of cDNA, 0.6 μL of primers, and 8.4 μL of SYBR® Green Pro Taq HS Premix (AG11701, Accurate Biology) employing a 7500 Real-Time PCR thermocycler (Applied Biosystem). The program steps for amplification were 95 °C for 30 s, 40 cycles of 95 °C for 3 s, and 60 °C for 30 s. Relative mRNA expression was calculated using the 2-∆∆CT method and β-actin as an endogenous control. The primers were synthesized by Takara Biomedical Technology and the primer sequences are listed in Table 1.

**Table 1:** Primers utilized in real-time qRT-PCR reactions

Gene	Forward primer (5′-3′)	Reverse primer (5′-3′)
cGAS	5′-AGGAAGCCCTGCTGTAACA CTTCT-3′	5′-AGCCAGCCTTGAATAGGTAGT CCT-3′
STING	5′-GGCGTCTGTATCCTGGAGT A-3′	5′-TAGACAATGAGGCGGCAGTTA T-3′
TNF-α	5′-ACTCCAGGCGGTGCCTATG T-3′	5′-GTGAGGGTCTGGGCCATAGA A-3′
IL-6	5′-CCACTTCACAAGTCGGAGG CTTA-3′	5′-CCAGTTTGGTAGCATCCATCAT TTC-3′
β-actin	5′-CATCCGTAAAGACCTCTATG CCAAC-3′	5′-ATGGAGCCACCGATCCACA-3′

### Flow cytometry

Flow cytometry was used to assess neuroinflammation after CVST as previously reported [36]. On day 3 after CVST and perfusion with pre-cooling PBS, the cerebral cortex tissues of mice were gently homogenized and passed through 70-um nylon cell strainers (15–1070, BIOLOGIX, China) in PBS. After centrifugation, the cell pellets were resuspended in 5 mL of 30% Percoll (P8370, Solarbio, China) and centrifuged at 700×*g* for 10 min. Cell pellets were harvested from the bottom of the tubes and washed once with 5 mL 1% BSA solution to block non-specific staining. Fresh single-cell suspensions were stained in the dark with directly labeled antibodies as follows: fluorescein isothiocyanate (FITC) anti-mouse CD45 (103107), phycoerythrin (PE) anti-mouse Ly6G (127607), allophycocyanin (APC) anti-mouse F4/80 (123115), or PerCP-Cy5.5 anti-mouse CD11b (101227). All fluorescent-labeled antibodies were purchased from BioLegend (San Diego, CA, USA). Finally, flow cytometry was performed using a FACS Aria III (BD CANTO, USA) and data were analyzed with FlowJo software.

### Statistical analysis

The data were expressed as the mean ± SEM and all statistical analyses were carried out utilizing SPSS 20.0 software (SPSS, IBM, Armonk, NY, USA). Comparison between three groups was determined by one-way analysis of variance (ANOVA) and followed by LSD test or Dunnett’s T3 test for the two groups’ comparison within the multiple groups. The *t*-test was used for comparison between the independent two groups. Correlation analysis was performed by Pearson test (if the data do not conform to a normal distribution, the logarithm is taken). Statistical significance was accepted when *P* < 0.05. Data were analyzed by investigators blinded to the identity of groups during the whole experiment.

## Results

### Dynamic changes of double-strand DNA (dsDNA) and its sensors cGAS and STING triggered by CVST

Mice were randomized to six groups containing a sham group and five CVST subgroups (at 6 h, 1 d, 3 d, 7 d, and 14 d post-CVST). The expression of dsDNA in the damaged cortex at indicated times was detected by IF staining. In sham controls, dsDNA expression was finite, mainly presenting a circular nuclear contour, as noted unanimously in the literature [9]; whereas, after the induction of CVST at 24 h and 3 days, the intensities of dsDNA notably elevated with increased distribution both in the cytoplasm and nucleus. Especially, arresting disintegration of the nucleus was observed at 3 days after CVST (Fig. 1A). The white frames in Fig. 1B denoted the selected brain cortex regions for the whole experiment. In line with the elevation of dsDNA accumulation post-CVST, by IF and qPCR, we demonstrated that enhanced expressions of cGAS and STING in the impaired cortex compared with the controls on day 3 following CVST and predominantly located in the cytoplasm (Fig. 1C and D). Simultaneously, the WB results suggested that compared to the sham controls, the protein expressions of cGAS and STING strikingly increased from 24 h and reached the summit at 3 days, and then maintained at a high level at 7 days post-CVST. Despite falling distinctly at 14 days, they were still higher than the sham groups (Fig. 1E and F). Importantly, by fluorescent double-labeling, we found that cGAS was mainly localized in cells abundant in aberrant or ectopic dsDNA, potently indicating that accumulation of self-derived or enthetic dsDNA in the cytosol may trigger the activation of the cGAS–STING pathway post-CVST (Fig. 1G).

### Cell localization of cGAS and STING after CVST

**Fig. 2:**
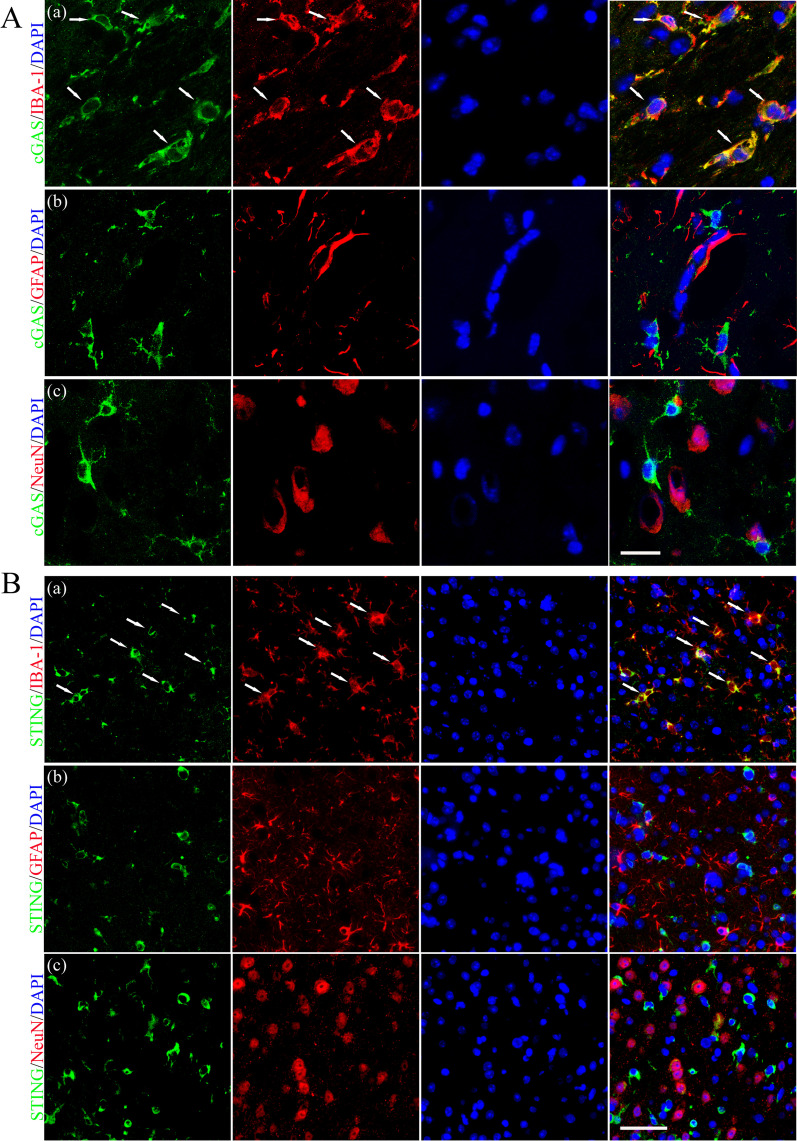
Cell localization of cGAS and STING after CVST. Immunofluorescent double staining demonstrated the cGAS and STING mainly localized in the microglia (IBA-1, **A**(**a**) and **B**(**a**), as indicated by the white arrows, respectively) but rarely in astrocytes (GFAP, **A**(**b**) and **B**(**b**)) and neurons (NeuN, **A**(**c**) and **B**(**c**)) at 3 d after CVST. **A** Scale bar = 20 um, **B** scale bar = 50 um

Immunofluorescent double staining showed that the cGAS and STING are mainly expressed in the activated microglia/macrophages (IBA-1^+^) but rarely in neurons (NeuN^+^) and astrocytes (GFAP^+^) at 3 d after CVST (Fig. 2A and B).

### Time course expression levels of NLRP3, GSDMD, GSDMD-C post-CVST

**Fig. 3:**
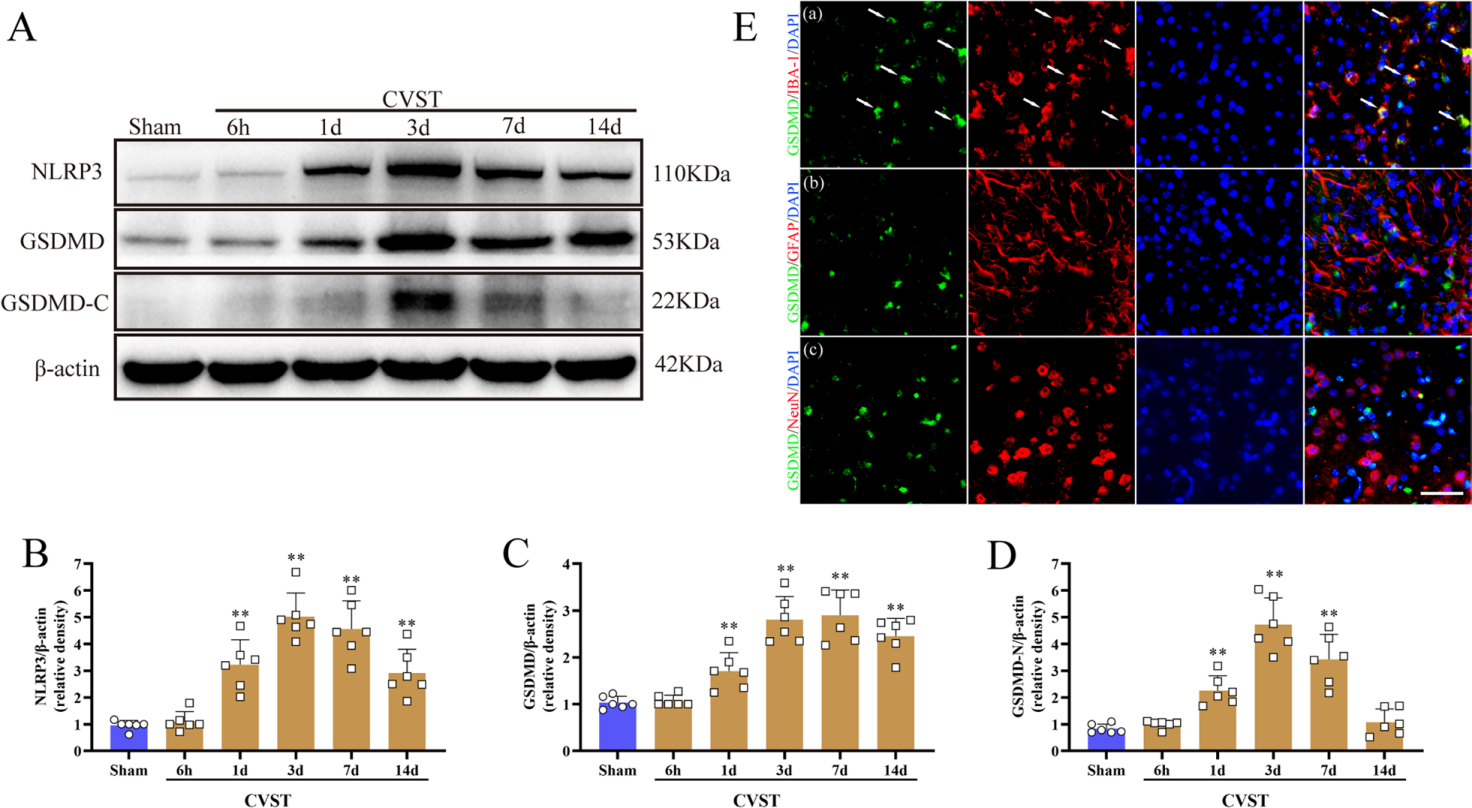
Time course expression of NLRP3, GSDMD and GSDMD-C in cerebral cortex tissues and cell localization of GSDMD after CVST. **A** Western blot assay of the temporal profiles of NLRP3, GSDMD, and GSDMD-C expressions from the injured cortex at 6 h, 1 d, 3 d, 7 d, and 14 d post-CVST. **B**–**D** Quantitative analysis for western blot assay. Bars represent mean ± SEM. **P < 0.01 vs Sham group. n = 6 per group. **E** Immunofluorescent double staining showed the GSDMD mainly localized in the microglia/macrophage (IBA-1, **B**(**a**), as indicated by the white arrows) but rarely in astrocytes (GFAP, **B**(**b**)) and neurons (NeuN, **B**(**c**)) at 3 d after CVST. Scale bar = 50 um

The results of WB revealed that compared to the sham groups, the prominent elevation of NLRP3 started at 24 h post-CVST and peaked at 3 days, and then gradually declined until 14 days (Fig. 3A and B). Concurrently, the expressions of GSDMD and GSDMD-C have analogous tendencies as those of NLRP3 (Fig. 3A, C and D). Notably, GSDMD C-terminal is generated simultaneously and in equal proportion with GSDMD-N terminal from the total GSDMD, which may be used as a marker of GSDMD-N production as the prior report [37, 38]. Besides, IF staining exhibited that GSDMD was predominantly localized in IBA-1^+^ microglia/macrophages in the cerebral cortex but hardly in neurons and astrocytes at 3 d post-CVST (Fig. 3Ea).

### RU.521 delivery alleviated apoptosis, neurodegeneration, and ameliorated neurobehavioral deficits post-CVST

**Fig. 4:**
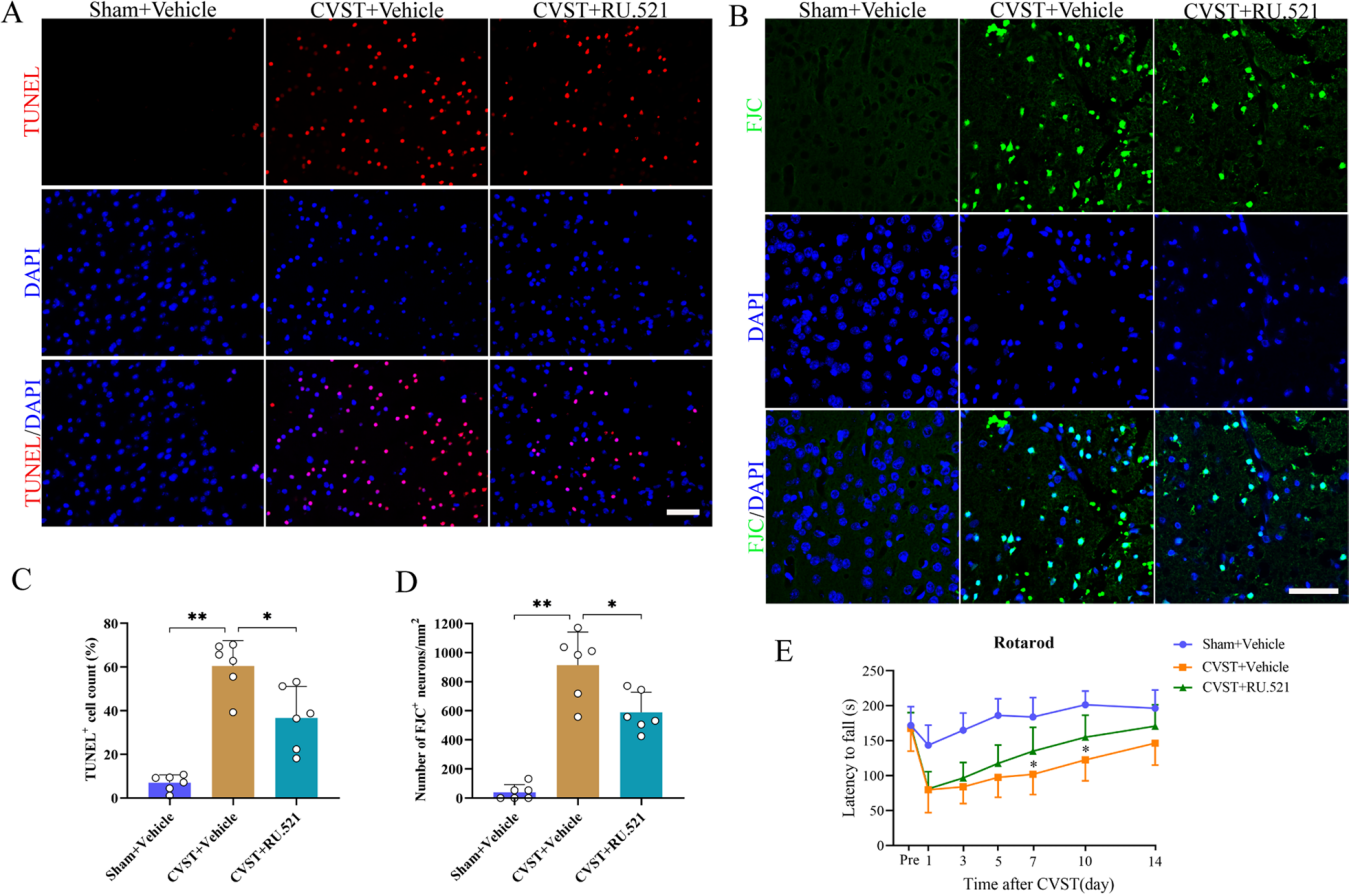
Effect of RU.521 on cortical neuronal injury and motor function after CVST. **A** Typical photomicrographs of TUNEL staining and **C** quantitative analyses at the indicated groups. n = 6 per group. **B** Representative pictures of FJC staining and **D** quantitative analyses in diverse groups. n = 6 per group. **E** Motor function was evaluated by rotarod test at pre-surgery and 1 d, 3 d, 5 d, 7 d, 10 d and 14 d post-operation. The number of mice in the Sham + vehicle group, CVST + vehicle group, and CVST + RU.521 group was 6, 8, and 9, respectively. ^**^P < 0.01, ^*^P < 0.05. Scale bar = 50 μm

Mice were randomly divided into three groups including sham + vehicle group, CVST + vehicle group and CVST + RU.521 treatment group. RU.521 is a small-molecule inhibitor of cGAS with potent selectivity, which can effectively restrain its dsDNA-dependent enzyme activity [12, 32]. At 3 days after CVST ictus, TUNEL-positive cells were markedly increased in the damaged cortex compared with the sham groups, nasal delivery of RU.521 significantly decreased the number of apoptotic cells (Fig. 4A and C). Additionally, by double fluorescence labeling, we found that TUNEL-positive cells co-localized mostly with NeuN, a neuronal marker, whereas hardly with IBA-1, a microglia/macrophage symbol (Additional file 2: Fig. S2). Synchronously, FJC staining revealed that the degenerated neurons were robustly augmented post-CVST, and RU.521 markedly reduced the number of dying neurons (Fig. 4B and D). Furthermore, compared with the sham groups, the CVST mice developed moderate motor deficits lasting for at least 10 days, as evidenced by the rotarod test (Fig. 4E). In contrast, the RU.521-treated mice exhibited significantly improved motor function relative to the CVST + vehicle group on day 7 and day 10 after CVST.

### RU.521 treatment ameliorated oxidative stress injury after at 3 d CVST

**Fig. 5:**
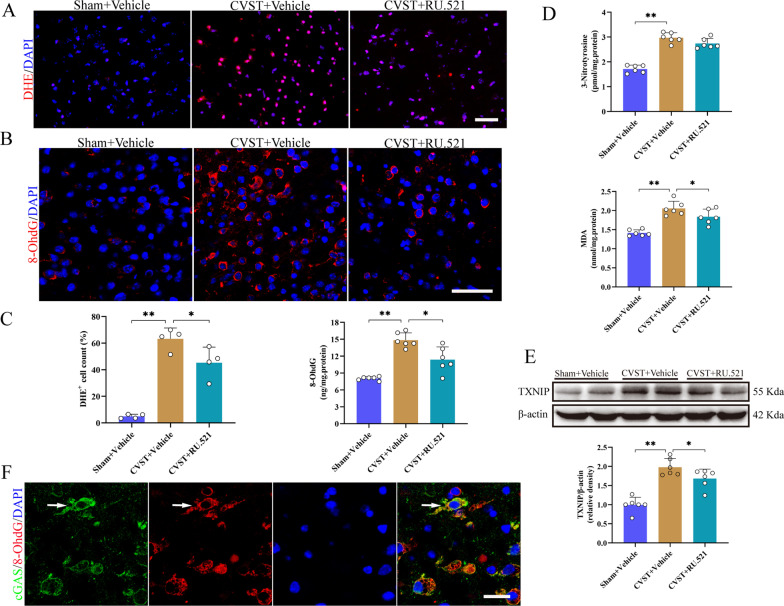
Effect of RU.521 on oxidative damage after CVST. **A** Typical photomicrographs of DHE staining in diverse groups. Scale bar = 50 μm. **B** Representative fluorescent staining of 8-OhdG in the indicated groups. Scale bar = 50 μm. **C**, **D** Quantitative analysis of different markers of oxidative damage (ROS, 8-OhdG, 3-nitrotyrosine, and MDA). Bars represent mean ± SEM. ^**^P < 0.01, ^*^P < 0.05. n = 4/6 per group. **E** Western blot assay and quantitative analysis of TXNIP in the damaged cortex post-CVST. **F** Immunofluorescent double staining demonstrated the enhanced 8-OhdG and cGAS exists an obvious co-localization relationship (as shown by white arrow). Scale bar = 20 μm

CVST induced eminently oxidative damage, as evidenced by DHE staining, IF, ELISA, and WB assays, demonstrating increases in the levels of ROS, 8-OHdG (a marker of DNA oxidative damage), MDA (an indicator of lipid peroxidation), 3-nitrotyrosine (an index of protein tyrosine nitration), and TXNIP when compared with those of the sham + vehicle groups (Fig. 5A–E). Contrarily, RU.521 administration partly counteracted the aforementioned variations and mitigated oxidative stress after CVST, which was basically in accord with the prior record [39]. Moreover, IF staining demonstrated that augmented 8-OhdG was noticeably co-localized with cGAS (Fig. 5F), implying that oxidized DNA may be strikingly involved in the activation of cGAS following CVST.

### Inhibition of STING signaling and NF-κb-related pro-inflammatory cytokines as well as inflammatory cells by RU.521 after CVST

**Fig. 6:**
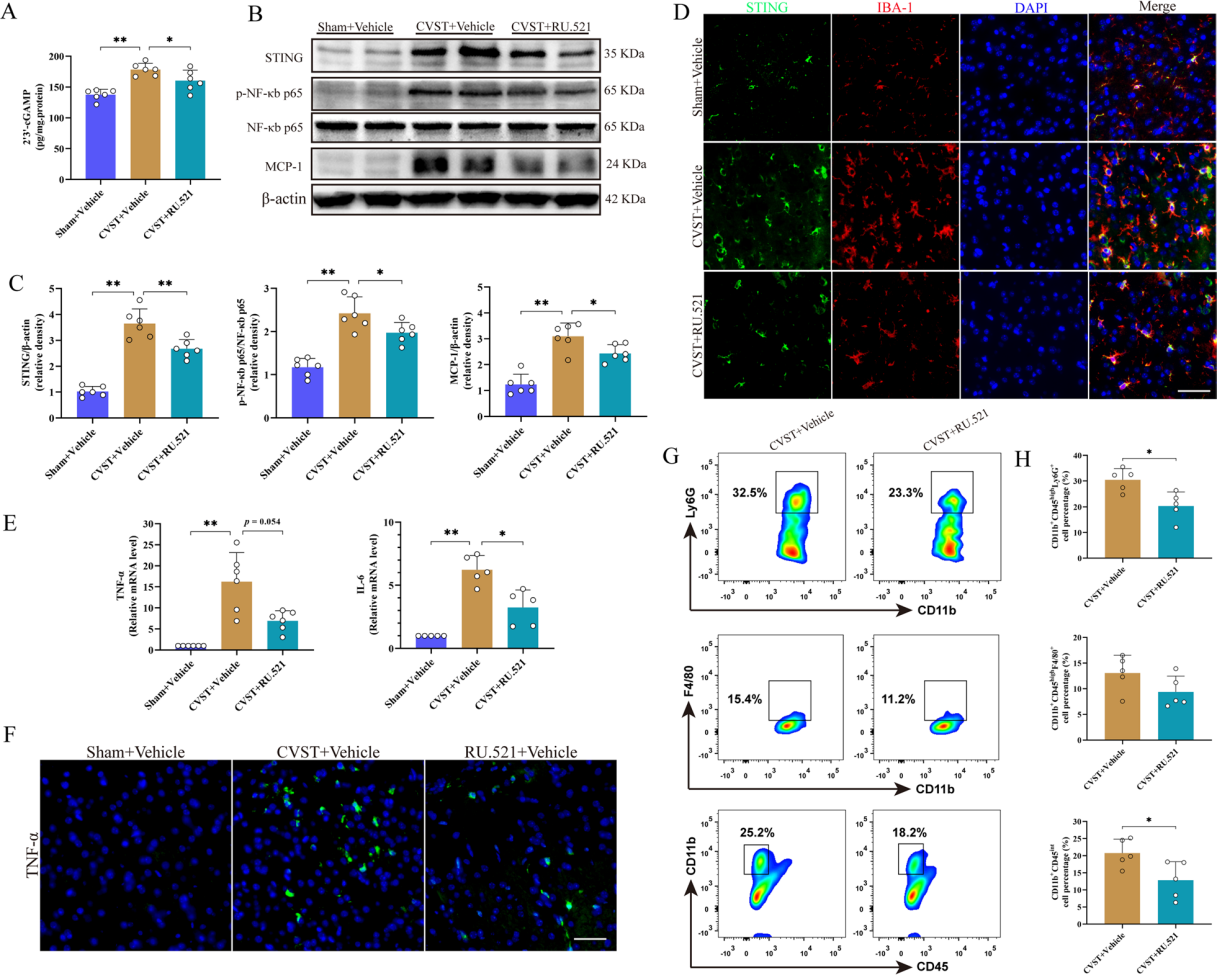
Effects of RU.521 on STING and related inflammatory factors as well as inflammatory cell infiltration after CVST. Elisa assay and quantitative analysis of 2′3′-cGAMP in diverse groups. n = 6 per group. **A** Representative western blot images of STING, p-NF-κb p65, NF-κb p65 and MCP-1. n = 6 per group. **B** Quantitative analysis of western blot assay. ^*^P < 0.05, ^**^P < 0.01. **C** Double immunofluorescence staining for STING (green) and microglia (IBA-1, red) in different groups. Scale bar = 50 μm. **D** Quantitative PCR analysis of cytokines (TNF-α and IL-6) on 3 days post-CVST. n = 5 to 6 per group. ^*^P < 0.05, ^**^P < 0.01. **E** Representative images of TNF-α expression in diverse groups. Scale bar = 50 μm. **F** Flow cytometry assay of brain-infiltrating immune cells including neutrophils (CD11b^+^CD45^high^Ly6G^+^), monocyte/macrophages (CD11b^+^CD45^high^F4/80^+^) and microglia (CD11b^+^CD45^int^) in the damaged cortex on 3 days after CVST. **G** Quantitative analysis of infiltrated immune cells. n = 5 per group. ^*^P < 0.05

We employed the ELISA to determine the concentration of 2′3′-cGAMP in the injured cerebral cortex and revealed that endogenous level of 2′3′-cGAMP, which is naturally synthesized by activated cGAS, was markedly augmented at 3 days after CVST compared to the sham group, confirming that cGAS is activated following CVST. In contrast, treatment with RU.521 significantly reduced the increased level of 2′3′-cGAMP (Fig. 6A). Besides, data from WB and/or PCR showed that the levels of STING and NF-κb phosphorylation along with downstream inflammatory factors (MCP-1, IL-6 and TNF-a), were highly induced in the brain cortex of vehicle-treated CVST animals, whereas RU.521 treatment partly reversed the induction of STING and relevant mediators (Fig. 6B, C and E). In concert with the above results, parallel alterations of STING and TNF-a in diverse groups were also proved by IF staining (Fig. 6D and F).

Next, we attempted to determine the effects of RU.521 on brain inflammatory cells after CVST via flow cytometry (Additional file 3: Fig. S3). We discovered that compared to the vehicle-treated controls, RU.521 delivery notably dampened neutrophils (CD11b^+^CD45^high^Ly6G^+^) infiltration and decreased the number of microglia (CD11b^+^CD45^int^) in the brain cortex on day 3 after CVST (Fig. 6G and H). A decrease of CVST-induced infiltration of monocyte/macrophages (CD11b^+^CD45^high^F4/80^+^) by RU.521 treatment was also noticed, while that was not statistically significant. To further verify the impact of RU.521 on the above inflammatory cells, we performed IHC analysis demonstrating that the number of neutrophils and microglia/macrophages was reduced by RU.521 treatment as indicated by Ly6G and Iba-1 immunoreactivity (Additional file 4: Fig. S4). These results indicate that RU.521 can suppress neuroinflammation via regulation of both resident microglia and infiltrating immune cells after CVST.

### Suppression of NLRP3 inflammasome and pyroptosis by RU.521 after CVST

**Fig. 7:**
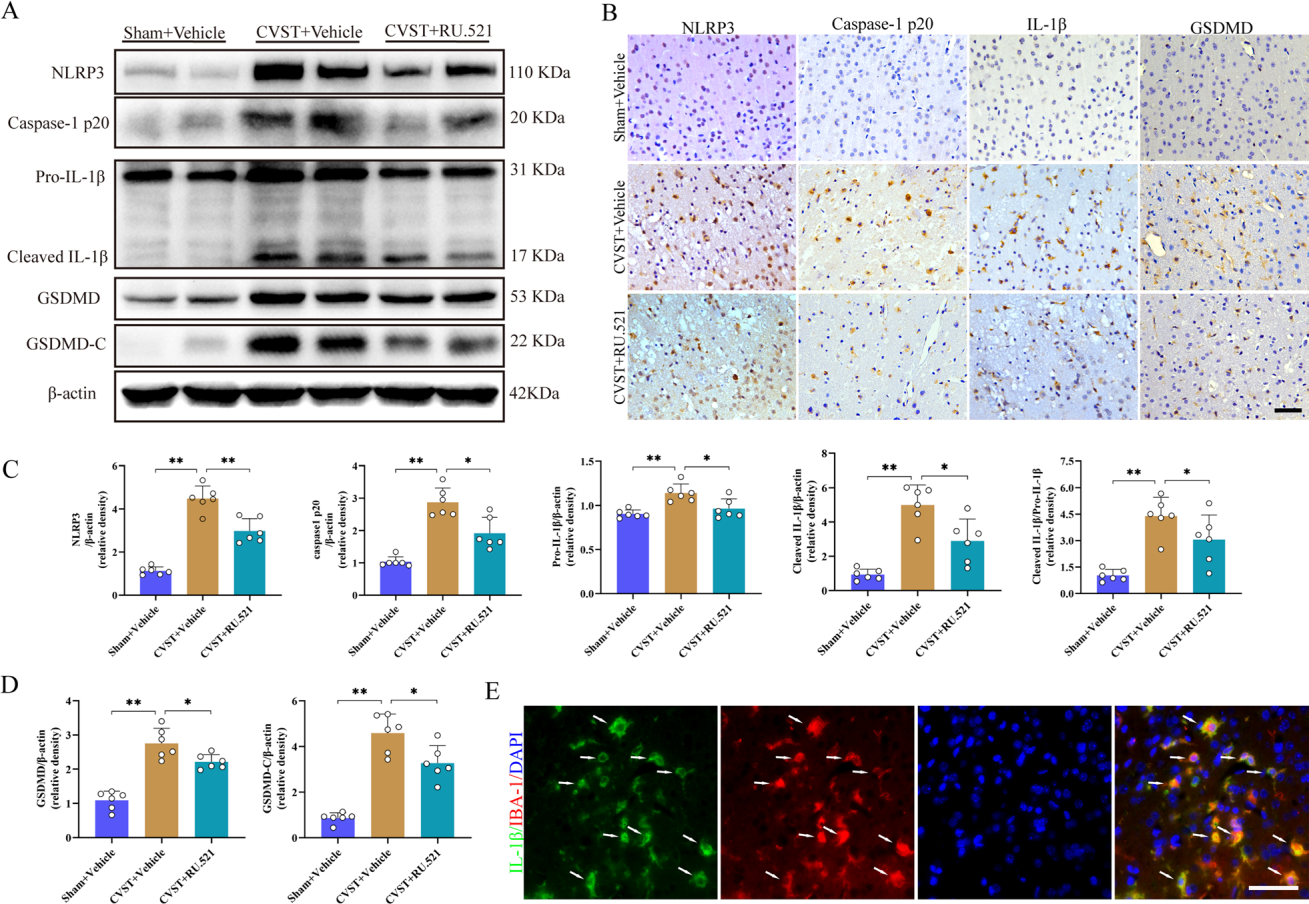
Effects of RU.521 on NLRP3 inflammasome and pyroptosis post-CVST. **A** Western blot assay for NLRP3, caspase-1 p20, pro-IL-1β, cleaved-IL-1β, GSDMD and GSDMD-C expressions from the impaired cortex at 3 days post-CVST. **B** Typical immunohistochemical staining for NLRP3, caspase-1 p20, IL-1β and GSDMD at diverse groups after CVST. Scale bar = 50 μm. **C**, **D** Quantitative analysis of western blot assay. ^*^P < 0.05, ^**^P < 0.01. n = 6. **E** Representative co-localization staining for IL-1β (green) and microglia (IBA-1, red) in the injured cortex as shown by white arrows, at 3 days after CVST. Scale bar = 50 μm

By WB and IHC (Fig. 7A–D), we observed that protein levels of NLRP3 inflammasome-related components (NLRP3, caspase-1 p20, pro-IL-1β, cleaved-IL-1β, cleaved-IL-1β/Pro-IL-1β), and pyroptosis executor GSDMD along with its cleaved C-terminal (GSDMD-C) were strikingly increased in the ischemic cortex after CVST in comparison with the sham-operated groups, whereas RU.521 delivery markedly repressed the aforementioned alterations. Additionally, a notable co-localization link existed between IL-1β and IBA-1 (Fig. 7E).

### Effect of STING on neuronal injury, neuroinflammation and pyroptosis after CVST

**Fig. 8:**
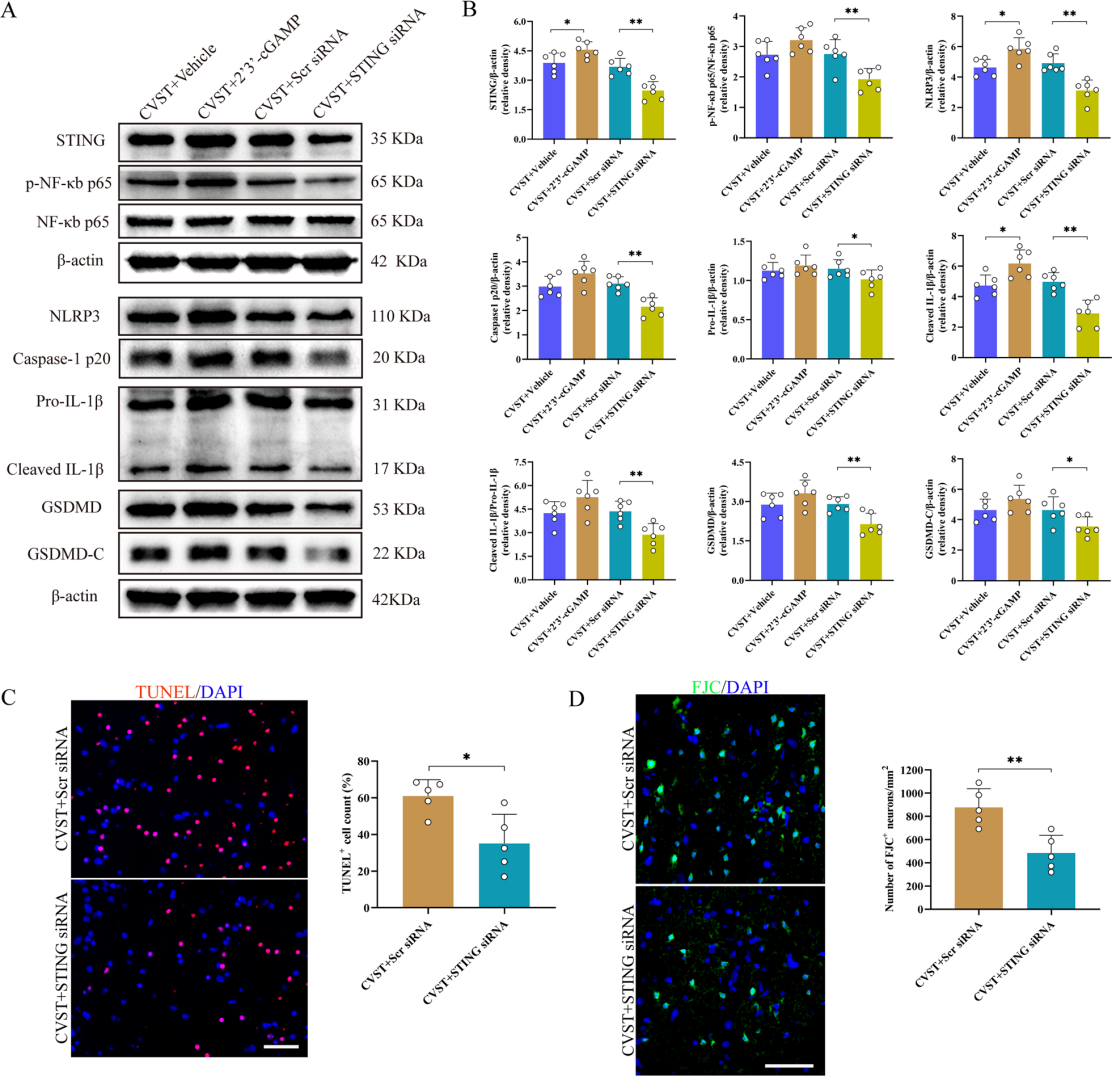
Effects of STING on NLRP3 inflammasome and pyroptosis as well as neuronal damage post-CVST. **A** Representative western blot images of STING, p-NF-κb p65, NF-κb p65, NLRP3, caspase-1 p20, pro-IL-1β, cleaved IL-1β, GSDMD, GSDMD-C at 3 days post-CVST. **B** Quantitative analysis of western blot assay. ^*^P < 0.05, ^**^P < 0.01. n = 6. **C** Representative microphotographs and quantitative analyses of TUNEL-positive cells (red). Scale bar = 50 μm. ^*^P < 0.05, n = 5. **D** Typical microphotographs and quantitative analyses of FJC-positive cells (green). Scale bar = 50 μm. ^**^P < 0.01, n = 5

To probe into the potential role of STING in the pathological process post-CVST, the specific STING agonist 2′3'-cGAMP and STING siRNA were utilized, separately. WB results indicated that in comparison with the CVST + vehicle group, the expression of STING was significantly increased after 2′3′-cGAMP delivery. In contrast, intraventricular injection of STING siRNA (The efficiency was confirmed by WB as shown in Additional file 5: Fig. S5) efficiently dropped the expression of STING when compared with the CVST + Scramble siRNA group (Fig. 8A and B). Analogously, the phosphorylation level of NF-κb was moderately upregulated by 2′3′-cGAMP compared to the CVST + vehicle group, while remarkably repressed by STING siRNA application. Additionally, we also demonstrated that the protein levels of NLRP3, caspase-1 p20, pro-IL-1β, cleaved-IL-1β, cleaved-IL-1β/Pro-IL-1β, GSDMD, and GSDMD-C were further enhanced after 2′3′-cGAMP delivery in comparison with the CVST + vehicle groups, whereas injection of STING siRNA markedly reduced the levels of aforementioned proteins when compared with the CVST + scramble siRNA groups (Fig. 8C and D). What’s more, delivery of STING siRNA also remarkably restrained cell apoptosis and decreased the counts of dying neurons in the CVST + scramble groups, as demonstrated by TUNEL and FJC staining (Fig. 8E and F).

## Discussion

**Fig. 9:**
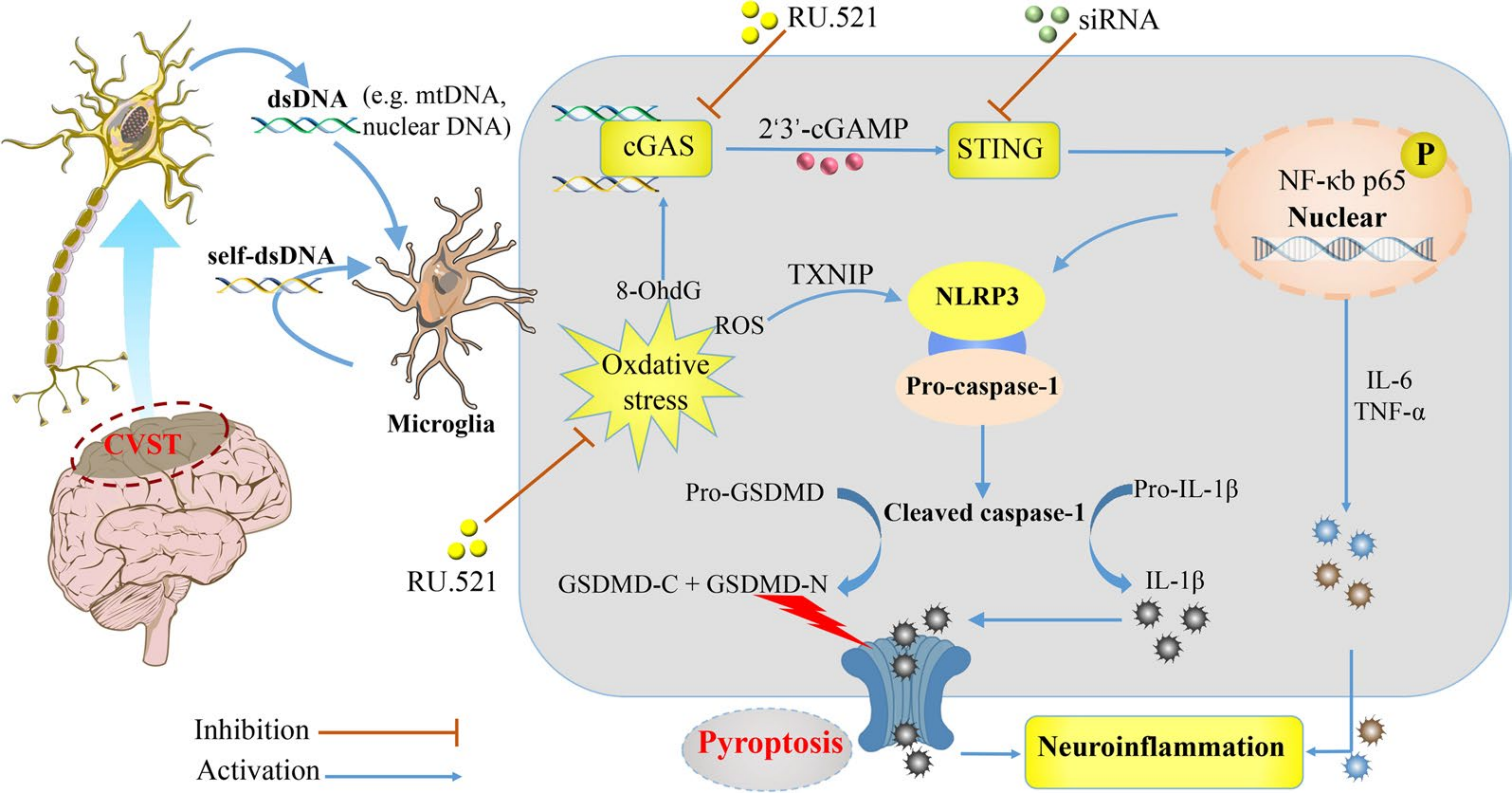
A schematic diagram displaying the pyroptosis and neuroinflammatory cascades on dsDNA recognition by microglia cGAS–STING axis during CVST. We demonstrated that suppression of dsDNA-sensing cGAS–STING pathway may exert neuroprotection and ameliorate the neuroinflammatory pathology of CVST, possibly by inhibiting NLRP3 inflammasome activation and GSDMD-mediated pyroptosis. In addition, concomitant oxidative stress may involve activation of cGAS and NLRP3 inflammasome through diverse mechanisms, ultimately contributing to the combustion of the neuroinflammatory process post-CVST

In this study, we found that cGAS was remarkably elevated in a CVST mouse model and contributed to neuroinflammation. Blockage of cGAS with a specific inhibitor, RU.521, strikingly alleviated inflammatory responses via inhibition of the levels of STING and NF-κb-related inflammatory cytokines, infiltration of immune cells, NLRP3 inflammasome activation, and GSDMD-sparked microglia pyroptosis. Furthermore, RU.521 treatment also ameliorated oxidative stress, neuronal death, and neurobehavioral performance post-CVST. Besides, we demonstrated that STING activation by 2′3′-cGAMP moderately intensified the NF-κb inflammatory cascade, NLRP3 inflammasome, and pyroptosis-related proteins. Conversely, the silencing of STING with siRNA counteracted this inflammatory process and improved the neuronal lesions. Overall, our results demonstrate that repression of the cGAS–STING pathway may be a potential therapeutic strategy to mitigate the neuroinflammatory pathology in CVST (Fig. 9).

Cytosolic dsDNA is a danger-associated molecular pattern that activates the inflammatory and immune responses. Components of endogenic dsDNA contain mitochondrial DNA (mtDNA) and disintegrated nuclear DNA [40, 41] that may be released in large quantities by necrotic neurons attributed to CVST ictus (Fig. 2A). cGAS has been identified as a critical cytosolic DNA sensor that detects dsDNA in a length-dependent while the sequence-independent manner [7], which is closely associated with neuroinflammation in the animal models of ischemic stroke [9, 42], amyotrophic lateral sclerosis [11], and traumatic brain injury [43].

Our results show that cGAS is activated in response to mislocalized dsDNA, triggering downstream pro-inflammatory signals mediated by the STING cascade. Also, oxidative stress has been reported to activate the cGAS signaling by facilitating the binding of cGAS to pathogenic mtDNA [42]. Enhanced hallmarks of oxidative stress (ROS, MDA, 3-nitrotyrosine and 8-OhdG) have been demonstrated in current and prior CVST-pertinent studies [44, 45]. More importantly, a noticeable co-localization has been observed between enhanced cGAS and 8-OhdG post-CVST which fits with the recent theory that 8-OhdG is a direct ligand of cGAS [46], and consequently, anti-8-OhdG therapy may be an effective route to regulate the cGAS–STING axis after CVST.

RU.521 is the most extensively utilized cGAS inhibitor that potently and selectively suppresses cGAS enzyme activity and downstream inflammatory events in mice and humans [32, 47]. It has also recently been shown to have anti-oxidant and anti-apoptotic properties [39]. Importantly, an original study targeting the cGAS–STING axis with intranasal delivery of the RU.521 demonstrated its neuroprotective roles in the brains of an experimental neonatal hypoxia–ischemia rat model [12]. Similarly, our data revealed that RU.521 also gives play to neuroprotection through indirect anti-inflammatory (as evidenced by lessened levels of STING and downstream inflammatory factors as well as reduced microglia and neutrophil counts) and direct anti-oxidative (as indicated by decreased levels of ROS, MDA, and 8-OhdG) mechanisms in a mouse CVST model.

Microglia are the resident immune cells in charge of immune defense and inflammation modulation in the CNS [27, 42]. Of note, elevated cGAS and STING are predominantly localized in the microglia of the impaired cortex following CVST, and coincident cell localization has been reported in cerebral ischemia/reperfusion (I/R) [9] and subarachnoid hemorrhage [48] models. In addition, convincing evidence verified that specific deletion of cGAS in microglia eminently attenuates I/R-induced neuroinflammation and subsequent brain insults [9, 42]. These findings suggest that targeting the microglia cGAS–STING pathway is a more precise approach to modulate neuroinflammation based on dsDNA recognition post-stroke.

Among the NLR family members, the NLRP3 inflammasome is the most highly expressed member in microglia [18, 49] and has recently been identified in the CVST model [5]. Aberrant NLRP3 inflammasome activity makes for uncontrolled inflammation and underlies diverse CNS disorders [16, 29, 36]. Mechanistically, activation of the NLRP3 inflammasome requires at least two processes [13, 15, 16]. One is the activation of the NF-κb pathway which promotes the synthesis of NLRP3 protein and IL-1β precursor protein, the other is to trigger the assembly of the inflammasome, such as the production of ROS or TXNIP [18]. TXNIP is a key regulatory protein that boosts oxidative stress and has recently been identified as an NLRP3 binding protein that activates the NLRP3 inflammasome [50, 51]. Our previous investigation, by co-immunoprecipitation, disclosed that TXNIP engages in NLRP3 activation after CVST [52]. The current study displayed that the levels of phosphorylated-NF-κb p65, ROS, and TXNIP are distinctly raised post-CVST, which may synergistically conduce to the activation of the NLRP3 inflammasome. Concurrently, RU.521 delivery notably counteracted the above alterations and deactivated the NLRP3 inflammasome evidenced by the decreased expressions of NLRP3 and cleaved caspase-1 p20. The data further verify the anti-oxidant and anti-inflammatory properties of RU.521 as previously mentioned.

Pyroptosis is a pro-inflammatory form of programmed cell death that is distinct from apoptosis [14, 53]. GSDMD is a newly identified pyroptosis executioner comprising the pore-forming N-terminal domain (GSDMD-N) and the auto-inhibited C-terminus (GSDMD-C) which can be dissociated by cleaved caspase 1 from its precursor driven by NLRP3 activation [15, 28, 54]. Upon cleavage, the unlocked GSDMD-N is immediately oligomerized and translocated to the plasma membrane, triggering cell rupture and the release of inflammatory factors, such as IL-1β [55]. One of the remarkable findings of our study was that we verified the occurrence of pyroptosis in a CVST mouse model and showed that RU.521 had a robust effect against NLRP3 inflammasome and pyroptosis-related molecules (including GSDMD, GSDMD-C, pro-IL-1β, cleaved IL-1β and cleaved IL-1β/pro-IL-1β). Momentously, we also noted that activated microglia are the primary subset of cells with GSDMD immunoreactivity that are responsible for pyroptosis, similar to other neuroinflammation-relational disorders including ischemic stroke [9], post-cardiac arrest brain injury [28], and spinal cord injury [23].

STING (also known as MYPS and MITA, encoded by TMEM173) is a transmembrane homodimer that localizes to the endoplasmic reticulum [56]. Upon binding to cGAS-catalyzed second messenger 2′3′-cGAMP, STING rapidly triggers the inflammatory cascade and cell death (e.g., pyroptosis, apoptosis, and lysosomal cell death) via NF-κb signaling [57] or other routes [7, 58]. The interplay of the cGAS–STING axis and NLRP3 has been recently unveiled. Gaidt et al. [20] reported that in human myeloid cells infected by enthetic pathogens, the excited cGAS–STING axis initiates potassium efflux by orchestrating lysosomal cell death, eventually triggering NLRP3 inflammasome activation and pyroptosis. Moreover, in an LPS-induced cardiac damage model, STING activation by LPS stimulus triggered ROS/TXNIP-dependent NLRP3 activation, and STING knockout strikingly decreased the expression of NLRP3 and ameliorated cell death (including apoptosis and pyroptosis) and cardiac function [59]. Comparably, a recent investigation also showed that the mtDNA–cGAS–STING axis is involved in LPS-induced acute lung injury by modulating NLRP3 and pyroptosis of macrophages, whereas cGAS or STING deficiency efficiently abrogates the inflammatory amplification [60]. Our data revealed that RU.521 treatment significantly inhibited neuron apoptosis and microglia pyroptosis along with improved neurologic impairments. Besides, exogenous 2′3'-cGAMP binds and activates STING to activate the phosphorylation of NF-κb and synchronously the NLRP3 inflammasome and resultant pyroptosis. Knockdown of STING reversed the trend as described above and suppressed neuroinflammation indicating that the cGAS–STING axis engages in the NLRP3-mediated inflammatory milieu after CVST.

The present study preliminarily elucidated that the cGAS–STING pathway conduces to neuroinflammation possibly involving activation of the NLRP3 inflammasome and accompanying microglia pyroptosis in a CVST mouse model. However, our study had several limitations. Firstly, it may be advantageous to use a microglia-specific cGAS or STING-knockout mouse model to curb inflammation in response to aberrant dsDNA. Secondly, the role of the classical STING–IRF3–Type I interferons pathway in CVST was not explored. Thirdly, there are no data that functionally link NLRP3 to the inflammasome activation and connect the inflammasome with neuroinflammation and functional impairment after CVST. Additionally, our experiments focused on the regulatory actions of cGAS and STING on neuroinflammation and did not investigate the specific molecular mechanisms (e.g., lysosomal cell death, potassium efflux and so on) linking STING and NLRP3 inflammasome in the CVST model, which warrants further investigation.

In summary, the current study shows that the cGAS–STING axis is upregulated after CVST in animal setting and contributes to neuroinflammation, in all probability involvement of the NLRP3 inflammasome activation and concomitant microglia pyroptosis. These findings identify the dsDNA-sensing cGAS–STING axis as a potential target for salvaging the neuroinflammatory pathology post-CVST.

## Supplementary Information

**Additional file 1: Figure S1. **Experimental design and animal grouping. CVST, cerebral venous sinus thrombosis; WB, western blot; PCR, polymerase chain reaction, TUNEL, terminal deoxynucleotidyl transferase dUTP nick end labeling; FJC, fluoro-Jade C; DHE, dihydroethidium; ELISA, enzyme-linked immunosorbent assay; IF, immunofluorescence; IHC, immunohistochemical staining; cGAS, cyclic guanosine monophosphate (GMP)–adenosine monophosphate (AMP) synthase; STING, stimulator of interferon gene; NLRP3, Nod-like receptor family pyrin domain-containing 3; GSDMD, gasdermin-D; i.c.v, intracerebralventricular injection; siRNA, small interfering ribonucleic acid; Scr siRNA, scramble siRNA.

**Additional file 2: Figure S2. **Cell localization of TUNEL positive cells after CVST. Double fluorescence labeling showing that the TUNEL positive cells mainly localized in the neurons (NeuN, A(c) and A(d), as indicated by the white arrows) but rarely in microglia (IBA-1, B(c) and B(d), as shown by triangular white arrows) at 3 days after CVST. Scale bar = 50 um.

**Additional file 3: Figure S3.** Gating strategy of brain-infiltrating immune cells including neutrophils (CD11b^+^CD45^high^Ly6G^+^), monocyte/macrophages (CD11b^+^CD45^high^F4/80^+^), and microglia (CD11b^+^CD45^int^) in the cerebral cortex of mice after CVST.

**Additional file 4: Figure S4.** Effects of RU.521 on inflammatory cells post-CVST. (A) Typical immunohistochemical staining of brain immune cells containing neutrophils (Ly6G^+^), monocyte/macrophages (CD68^+^) and microglia (IBA-1^+^) in the damaged cortex of diverse groups at 3 days following CVST. Scale bar = 50 μm. (B) Quantitative analysis of the above immune cells. Bars represent mean ± SEM (n = 4 per group). ^**^P < 0.01, ^*^P < 0.05.

**Additional file 5: Figure S5. **Efficiency of siRNA-mediated knockdown of STING in injured cerebral cortex post-CVST. A) Typical micrographs of STING expression in CVST + Scr siRNA group and CVST + STING siRNA group at 3 days after CVST. Scale bar = 50 μm. B) Western blot assay and quantitative analyses of STING protein after the delivery of STING siRNA at 3 days post-CVST. Bars represent mean ± SEM (n = 4 per group). ^**^P < 0.01, ^*^P < 0.05.

## Data Availability

The datasets of the current study are available from the corresponding author on reasonable request.

## Abbreviations

CVST: Cerebral venous sinus thrombosis
cGAS: Cyclic GMP-AMP synthase
STING: Stimulator of interferon gene
NLRP3: Nod-like receptor family pyrin domain-containing 3
GTP: Guanosine triphosphate
ATP: Adenosine triphosphate
2′3′–cGAMP: Cyclic GMP-AMP
dsDNA: Double-stranded DNA
DAMP: Damage-related molecular pattern
NF-κB: Nuclear factor kappa-B
ASC: Apoptosis-associated speck-like protein containing CARD
ROS: Reactive oxygen species
TXNIP: Thioredoxin-interacting protein
GSDMD: Gasdermin-D
CNS: Central nervous system
SSS: Superior sagittal sinus
FeCl_3_: Ferric chloride
O.C.T.: Optimal cutting temperature
DMSO: Dimethylsulfoxide
DHE: Dihydroethidium
TUNEL: Terminal deoxynucleotidyl transferase dUTP nick-end labeling
FJC: Fluoro-Jade C
IF: Immunofluorescence
IHC: Immunohistochemistry
WB: Western blot
ELISA: Enzyme-linked immunosorbent assay
siRNA: Small interfering RNA
PBS: Phosphate-buffered saline
EDTA: Ethylene diamine tetraacetic acid
IL-1β: Interleukin-1 beta
IL-6: Interleukin-6
TNF-α: Tumor necrosis factor alpha
MCP-1: Monocyte chemoattractant protein-1
8-OHdG: 8-Hydroxy-2-deoxyguanosine
3-NT: 3-Nitrotyrosine
MDA: Malondialdehyde
qPCR: Quantitative real-time polymerase chain reaction
FITC: Fluorescein isothiocyanate
PE: Phycoerythrin
APC: Allophycocyanin
ANOVA: One-way analysis of variance
