# Supplementary figures and images for: Activating cGAS–STING axis contributes to neuroinflammation in CVST mouse model and induces inflammasome activation and microglia pyroptosis
Source: J Neuroinflammation. 2022 Jun 10;19:137. doi: 10.1186/s12974-022-02511-0 (PMC9188164; doi:10.1186/s12974-022-02511-0)

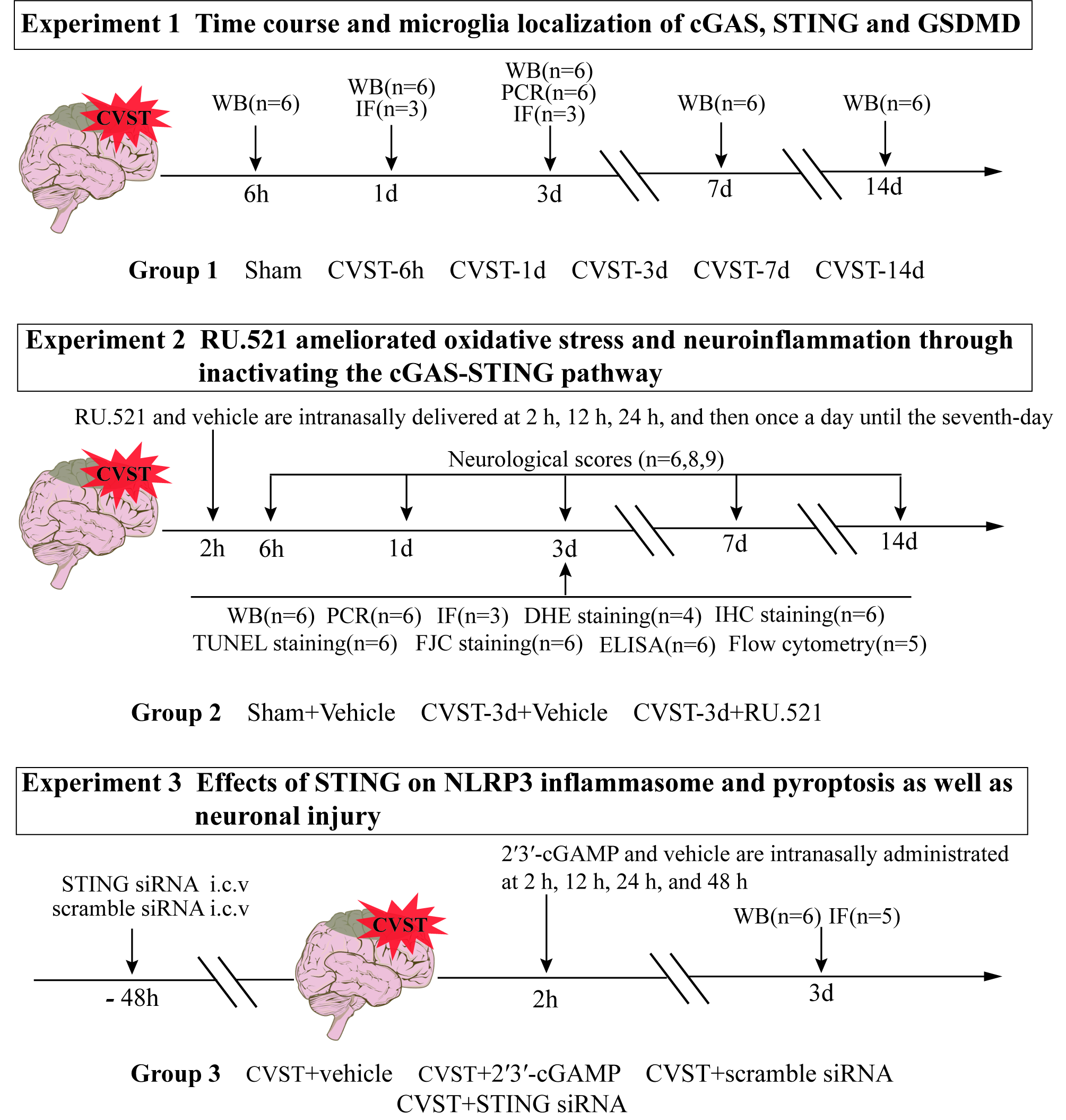

Supplement: Supplementary file 1 — Additional file 1: Figure S1. Experimental design and animal grouping. CVST, cerebral venous sinus thrombosis; WB, western blot; PCR, polymerase chain reaction, TUNEL, terminal deoxynucleotidyl transferase dUTP nick end labeling; FJC, fluoro-Jade C; DHE, dihydroethidium; ELISA, enzyme-linked immunosorbent assay; IF, immunofluorescence; IHC, immunohistochemical staining; cGAS, cyclic guanosine monophosphate (GMP)–adenosine monophosphate (AMP) synthase; STING, stimulator of interferon gene; NLRP3, Nod-like receptor family pyrin domain-containing 3; GSDMD, gasdermin-D; i.c.v, intracerebralventricular injection; siRNA, small interfering ribonucleic acid; Scr siRNA, scramble siRNA. [file 12974_2022_2511_MOESM1_ESM.tif]

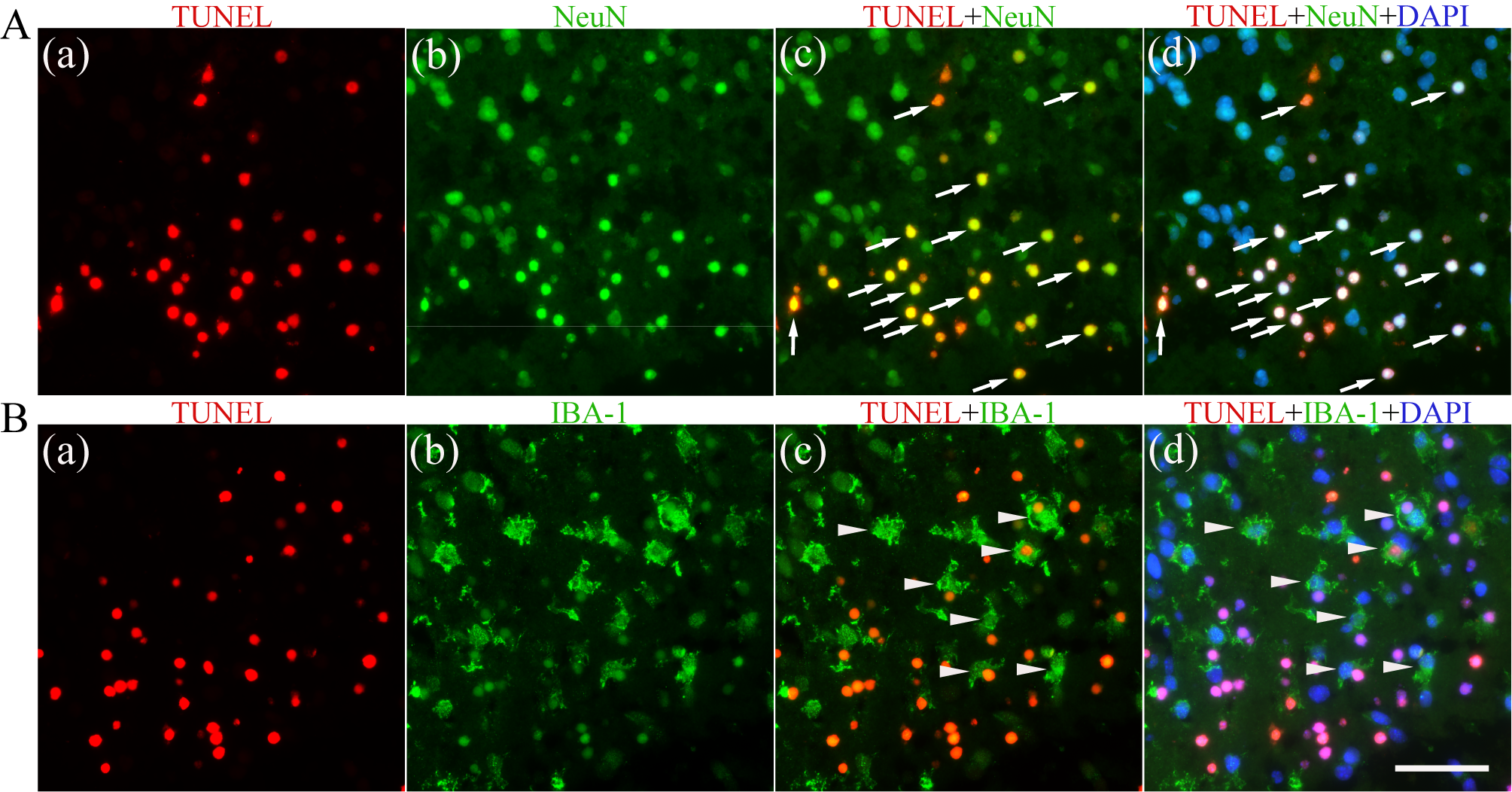

Supplement: Supplementary file 2 — Additional file 2: Figure S2. Cell localization of TUNEL positive cells after CVST. Double fluorescence labeling showing that the TUNEL positive cells mainly localized in the neurons (NeuN, A(c) and A(d), as indicated by the white arrows) but rarely in microglia (IBA-1, B(c) and B(d), as shown by triangular white arrows) at 3 days after CVST. Scale bar = 50 um. [file 12974_2022_2511_MOESM2_ESM.tif]

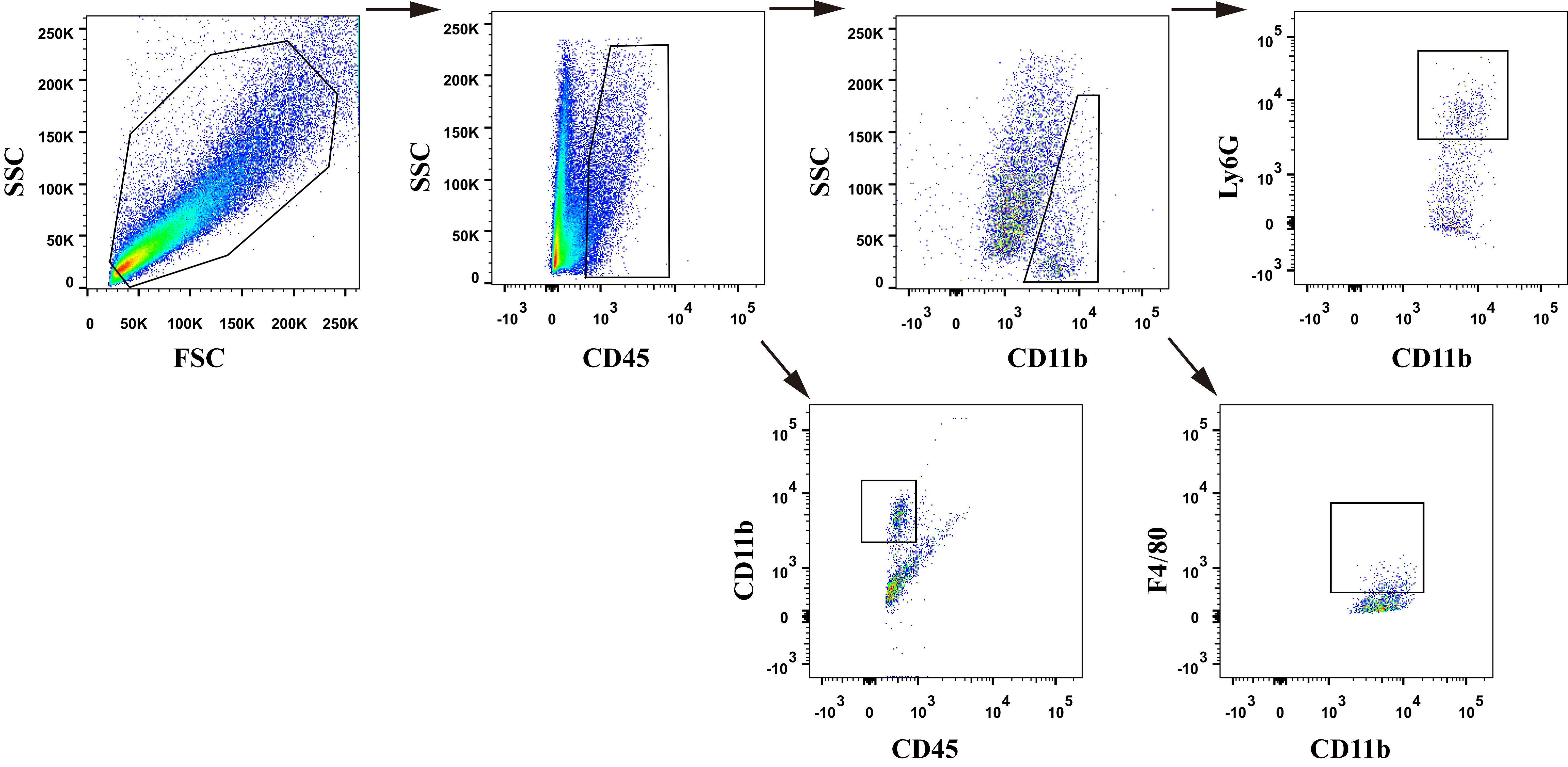

Supplement: Supplementary file 3 — Additional file 3: Figure S3. Gating strategy of brain-infiltrating immune cells including neutrophils (CD11b+CD45highLy6G+), monocyte/macrophages (CD11b+CD45highF4/80+), and microglia (CD11b+CD45int) in the cerebral cortex of mice after CVST. [file 12974_2022_2511_MOESM3_ESM.tif]

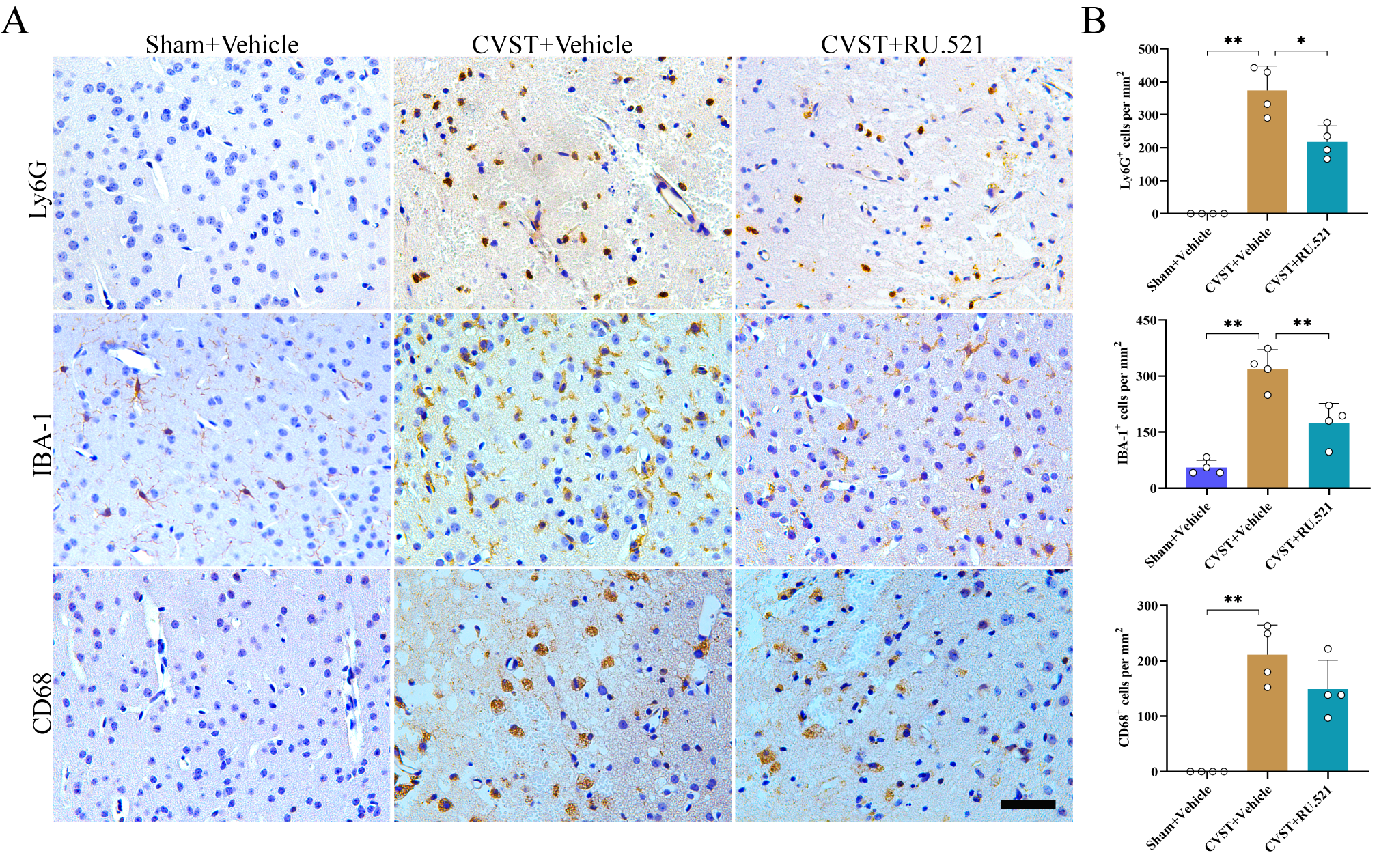

Supplement: Supplementary file 4 — Additional file 4: Figure S4. Effects of RU.521 on inflammatory cells post-CVST. (A) Typical immunohistochemical staining of brain immune cells containing neutrophils (Ly6G+), monocyte/macrophages (CD68+) and microglia (IBA-1+) in the damaged cortex of diverse groups at 3 days following CVST. Scale bar = 50 μm. (B) Quantitative analysis of the above immune cells. Bars represent mean ± SEM (n = 4 per group). **P < 0.01, *P < 0.05. [file 12974_2022_2511_MOESM4_ESM.tif]

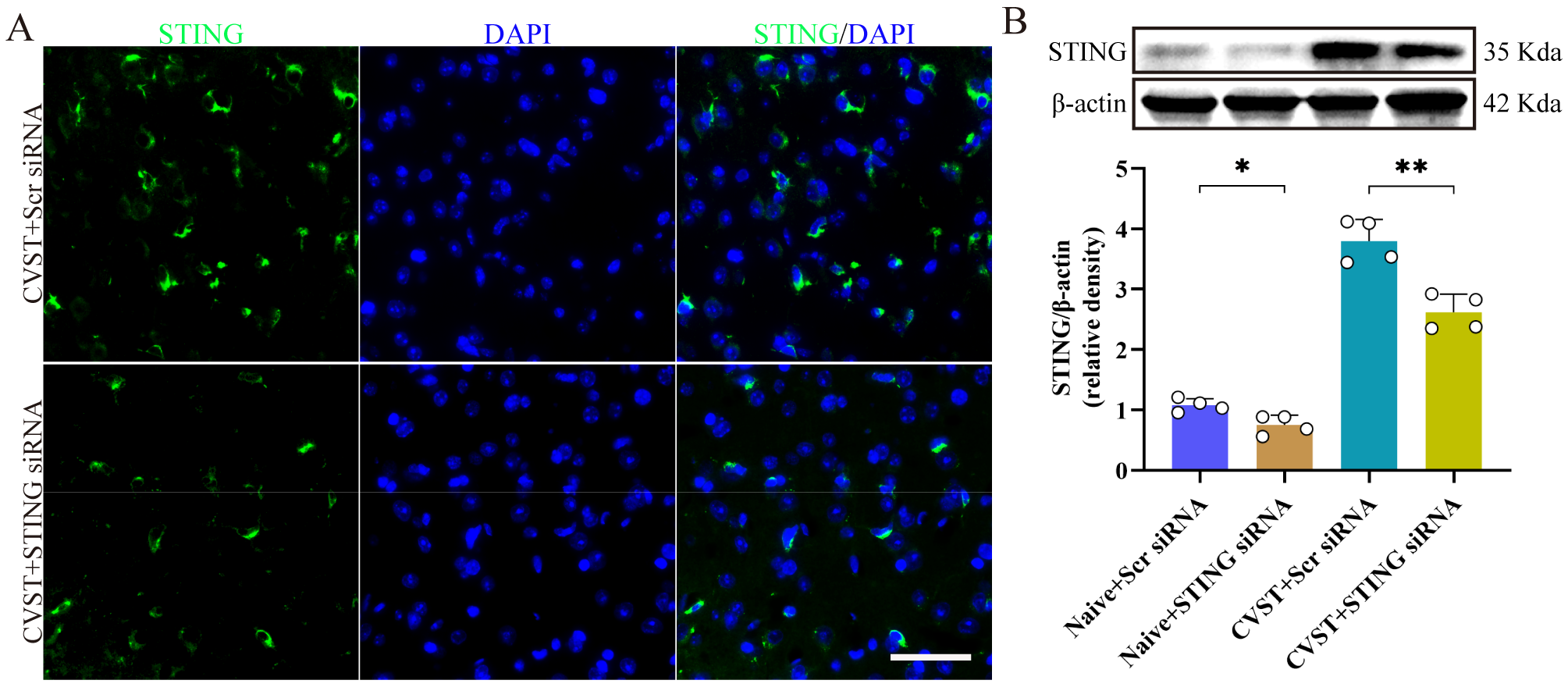

Supplement: Supplementary file 5 — Additional file 5: Figure S5. Efficiency of siRNA-mediated knockdown of STING in injured cerebral cortex post-CVST. A) Typical micrographs of STING expression in CVST + Scr siRNA group and CVST + STING siRNA group at 3 days after CVST. Scale bar = 50 μm. B) Western blot assay and quantitative analyses of STING protein after the delivery of STING siRNA at 3 days post-CVST. Bars represent mean ± SEM (n = 4 per group). **P < 0.01, *P < 0.05. [file 12974_2022_2511_MOESM5_ESM.tif]
